# Targeting HIC1/TGF-β axis-shaped prostate cancer microenvironment restrains its progression

**DOI:** 10.1038/s41419-022-05086-z

**Published:** 2022-07-19

**Authors:** Tianqi Wu, Wenfeng Wang, Guohai Shi, Mingang Hao, Yingying Wang, Mengfei Yao, Yongqiang Huang, Leilei Du, Xingming Zhang, Dingwei Ye, Xiaojie Bian, Jianhua Wang

**Affiliations:** 1 Cancer Institute, Shanghai Urological Cancer Institute, Fudan University Shanghai Cancer Center, Fudan University, Shanghai, 200032 P. R. China; 2 Department of Radiotherapy, Renji Hospital, School of Medicine, Shanghai Jiao Tong University, Shanghai, 200127 P. R. China; 3 Department of Urology, Fudan University Shanghai Cancer Center, Fudan University, Shanghai, 200032 P. R. China; 4 Department of Cancer Biology, University of Cincinnati College of Medicine, Cincinnati, OH 45267-0559 US; 5 Shandong Provincial Key Laboratory of Animal Cell and Developmental Biology, School of Life Sciences, Shandong University, Qingdao, 266237 P. R. China; 6 Department of Nuclear Medicine, Zhongshan Hospital, Fudan University, Shanghai, 200032 P. R. China; 7 School of Medicine, Anhui University of Science & Technology, Huainan, 232001 P. R. China

**Keywords:** Prostate cancer, Cancer microenvironment

## Abstract

Prostate cancer (PCa) is a malignant tumor that seriously threatens men’s health worldwide. Recently, stromal cells in the tumor microenvironment (TME) have been reported to contribute to the progression of PCa. However, the role and mechanism of how PCa cells interact with stromal cells to reshape the TME remain largely unknown. Here, using a spontaneous prostate adenocarcinoma (PRAD) model driven by the loss of *Pten* and *Hic1*, we found that M2 macrophages markedly infiltrated the stroma of *Pten* and *Hic1* double conditional knockout (*dCKO*) mice compared with those in control (*Ctrl*) mice due to higher TGF-β levels secreted by HIC1-deleted PCa cells. Mechanistically, TGF-β in TME promoted the polarization of macrophages into “M2” status by activating the STAT3 pathway and modulating *c-Myc* to upregulate CXCR4 expression. Meanwhile, TGF-β activated the fibroblasts to form cancer-associated fibroblasts (CAFs) that secrete higher CXCL12 levels, which bound to its cognate receptor CXCR4 on M2 macrophages. Upon interaction with CAFs, M2 macrophages secreted more CXCL5, which promoted the epithelial-mesenchymal transition (EMT) of PCa via CXCR2. Moreover, using the TGF-β receptor I antagonist, galunisertib, significantly inhibited the tumor growth and progression of the TRAMP-C1 cell line-derived subcutaneous tumor model. Finally, we confirmed that the stromal microenvironment was shaped by TGF-β in HIC1-deficient PCa and was associated with the progression of PCa.

## Introduction

Prostate cancer (PCa) is the most common malignant tumor in men in the United States [1, 2]. In recent years, the incidence and age-standardized mortality rates of PCa have also increased in mainland China [3]. Advanced PCa presents invasive features and patients usually suffer from distant metastasis, which is currently the leading cause of PCa-related death in men [4]. Due to the immunosuppressive tumor microenvironment (TME) of PCa, it is difficult to improve the response of immunotherapy [5]. Therefore, understanding the mechanism of immunosuppressive TME is critical to identifying novel therapeutic targets for PCa.

TME contains complex components of various non-tumor cells, such as infiltrating immune cells, fibroblasts, endothelial cells, and extracellular matrix, as well as multiple signaling molecules, including cytokines and chemokines [6]. Increasing evidence indicates that interactions between these cellular constituents in the TME play a central role in the development of malignancies. Among these cells, M2-type tumor-associated macrophages (TAMs) and cancer-associated fibroblasts (CAFs) are the main components of TME with immunosuppressive function [7]. Notably, M2-polarized TAMs, typically characterized by classical markers, such as CD206, CD163, and CD204, play a vital role in promoting oncogenesis and metastasis of tumors. They also inhibit antitumor immune responses mediated by T cells, and stimulate tumor angiogenesis and subsequent tumor progression [8]. Activated CAFs, which are phenotypically identified by α-SMA, fibroblast activation protein (FAP), and PDGFRα, secrete multiple chemokines, cytokines, and other factors [9]. TGF-β was reported to modulate the activation of fibroblasts in the TME [10].

Coincidentally, M2-type macrophages tend to appear in CAF-enriched areas [11, 12], suggesting close interactions between these two cell types. In neuroblastoma, TAMs settle in the vicinity of the CAF area and enhance reactivity between the two; Akt, STAT, and WNT signals are activated both in TAMs and CAFs [12]. CAFs derived from PCa prompt monocytes to migrate toward the tumor area and promote their transition to M2 phenotype macrophages by secreting IL6 and SDF1 [13]. CAFs induce an increased level of PD-1 expressed by M2-type macrophages, which not only inhibits the phagocytosis of macrophages but also inhibits T cell infiltration and proliferation, thereby promoting immune tolerance [14, 15]. Therefore, more and more researchers have proposed that targeting TAMs and CAFs may become a new direction for the treatment of malignant tumors. Some immune checkpoint inhibitors are already being developed, such as CD47-SIRPα blockade monoclonal antibodies targeting macrophages [16]. Our previous research proved that targeting the receptor of CXCL14 (GPR85) on the surface of CAFs, can inhibit the metastasis of breast cancer (BrCa). GPR85 may become a potential immune checkpoint for metastatic BrCa [17]. However, the molecular mechanisms underlying the crosstalk between TAMs and CAFs have not been elucidated, identifying the key cytokines and receptors that promote cancer metastasis in TME can provide new targets to improve immune responses and survival of patients.

Hypermethylated in cancer 1 (HIC1) is located on chromosome 17p13, which encodes a sequence-specific transcriptional repressor that belongs to the BTB/POZ and C2H2 zinc finger family [18]. The N-terminal BTB/POZ domain of HIC1 is responsible for protein-protein interactions, and the C-terminal zinc finger domains contribute to sequence-specific binding to an HIC1-responsive element (HiRE) through a TGCC (A/C) core motif [19]. As HIC1 is usually hypermethylated or deleted in several types of human tumors and promotes tumor progression, it is now known as a tumor growth regulator and tumor repressor [20–22]. Constitutive knockout of *Hic1* often leads to embryonic lethality [23], whereas heterozygous mice often develop various spontaneous malignant tumors [24]. Recently, we found that HIC1-deficiency reshapes TME by activating CAFs to promote migration and invasion of BrCa [17], suggesting that HIC1 may be an important switch regulating the immunosuppressive status of TME. However, the mechanism of HIC1 modulates the TME of PCa remains unclear.

In the present study, using a spontaneous PRAD model driven by the loss of *Pten* and *Hic1*, we found that M2 macrophages and CAFs markedly infiltrated the prostate stroma due to higher TGF-β levels secreted by HIC1-deleted PCa cells. Our goal was to explore the function of the HIC1/TGF-β axis in remodeling TME and to identify some important molecules involved in this pathological process. Finally, we confirmed that targeting HIC1/TGF-β axis in TME of PCa may reverse the malignant development of PCa.

## Materials and methods

### Generation of conditional knockout mice and tissue preparation

As described in our previous research [25], double conditional knockout mice were generated by crossing *Hic1*^*flox/flox*^ and *Pten*^*flox/flox*^ mice with *PB-Cre* mice in which Cre recombinase expression was driven by prostate-specific rat probasin promoter (Pb), which was only activated in the prostate epithelium. The Cre-positive; *Pten*^*flox/flox*^*; Hic1*^*flox*^^*/flox*^ mice were designated as *dCKO*, which were identified as the test group; littermates, Cre-positive; *Pten*^*flox/flox*^ mice, were designated as the *Pten*^−/−^ control group (*Ctrl*). These genotypes were confirmed by PCR-based analysis of the tail DNA (Supplementary Fig. S1a). Genotyping was based on PCR analysis of genomic DNA isolated from the tails or toes of mice using the Mouse Genotyping Kit (Cat. No. KK7352, Roche, Mannheim, Germany). Primers used for PCR-based genotyping are listed in the supplementary materials. All mice were maintained under specific pathogen-free (SPF) conditions. The experimental procedures followed the Shanghai Jiao-Tong University School of Medicine Committee for the Use and Care of Animals (Protocol number: A-2016-015).

Samples of prostate tissue were carefully separated from the mice and washed clean. The other tissues were separated quickly before fixation or frozen. Blood was drawn from the left ventricle of the mouse and stored in a refrigerator at −80 °C.

### Cell culture

The human PCa cell lines PC3, LNCap, LNCap C4-2B, LNCap C4-2, DU145, human normal prostate epithelial cell line RWPE-1, and mouse macrophage cell line RAW264.7, were obtained from American Type Culture Collection (ATCC) and cultured according to standard protocols from the ATCC website. Primary human peripheral blood mononuclear cells (PBMCs) were separated from the venous blood of healthy subjects. Primary CAFs of the prostate were isolated from patients treated with radical prostatectomy and normal fibroblasts (NFs) of the prostate were isolated from bladder cancer patients treated with radical surgeries. Additional details of primary cell separation and culture are described in the Supplemental data. The cells were cultured in a 37 °C water-saturated 5% CO_2_ atmosphere.

### Agilent cDNA microarray and analysis

An Agilent SurePrint G3 Human Gene Expression 8 × 60 K v2.0 Microarray was used in this experiment. RNA extraction, quality control, microarray hybridization, data extraction, and analysis were performed by Oebiotech Biotechnology Corporation, according to Agilent protocols. Hierarchical clustering was generated by R software (4.10) and R Studio (https://www.rstudio.com/). KEGG pathway analysis was enriched using the public tool DAVID 6.7 (https://david-d.ncifcrf.gov/tools.jsp) and generated with R Studio. Gene set enrichment analysis (GSEA) was performed using the GSEA software (v4.2.3). The microarray dataset was deposited in the NCBI Gene Expression Omnibus (GEO) database (GSE141642).

### Enzyme-linked immunosorbent assay (ELISA)

Quantification of TGF-β, CXCL12, and CXCL5 secretion levels in the supernatant of cells or mouse serum was performed using an ELISA kit (human TGF-β: Cat. No. ELH038; human CXCL12, Cat. No. ELH179; FMC Biology; mouse TGF-β: Cat. No. VAL611; NOVUS, human CXCL5: Cat. No. DX000, mouse CXCL5: Cat. No. MX000; R&D Systems, Minneapolis, MN). All experiments were repeated three times.

### Real-time cell analysis (RTCA) migration test

Migration of RAW264.7 cells was performed using the xCELLigence RTCA-DP system (Roche) according to published protocols^21^. The xCELLigence System allows real-time cell analysis (RTCA) by using the RTCA DP instrument equipped with a CIM-Plate 16. Briefly, we added 160 μL of PCa cell-conditioned medium (CM) to the low chamber of the CIM-Plate 16, followed by 30 μL of serum-free medium (SFM) into the upper chamber. Then, 4 × 10^4^ cells per well were resuspended in 100 μL of SFM and loaded into the upper chamber. The CIM-Plate 16 containing the cells was placed into the RTCA DP Analyzer inside the incubator at 37 °C for 48 h, and the cells that migrated to the lower chamber were monitored.

### Luciferase reporter assays and chromatin immunoprecipitation (ChIP)

*TGFB1* promoters and truncated constructs were synthesized from Obio Technology (Shanghai, China). *CXCR4* promoters were generated and preserved by our group [25]. All constructs and their mutants were inserted into the pGL3-basic reporter gene vector. The ChIP assay was performed according to the manufacturer’s protocol (Cat. No. CA52590, Millipore). Additional details are provided in Supplemental data.

### Hematoxylin–eosin (H&E) and immunohistochemistry (IHC) staining

Mouse prostates and lymph nodes were fixed in 4% formaldehyde overnight at room temperature and embedded in paraffin. Sections of 4 μm thickness were used for H&E and IHC staining. Samples were baked at 65 °C overnight, then de-paraffinized by two 10-min extractions in 100% xylene, followed by five min each of descending grades of alcohol (100, 95, and 75%). Samples were then washed briefly with phosphate-buffered saline (PBS) before being boiled for antigen retrieval in 10 mM sodium citrate buffer (pH 6.0) or EDTA (pH 8.0) for 15 min. For immunohistochemistry, sections were pre-treated with 3% hydrogen peroxide for 15 min before blocking.

Blocking was performed with 1% normal fetal bovine serum in PBS for 30 min at room temperature, followed by primary antibody incubation overnight at 4 °C. After washing away the primary antibodies, 3,3′-diaminobenzidine (DAB) was used as the substrate of the primary antibody for the immune enzymatic method, according to the manufacturer’s instructions (Cat. No. DA1010; Solarbio Life Science, Beijing, China).

The primary antibodies used were as follows: CK8 (Cat. No. ab59400; Abcam, Cambridge, UK), AR (Cat. No. sc-7305; Santa Cruz, Dallas, TX), HIC1 (Cat. No. bs-15485R; Bioss, Boston, MA), α-SMA (Cat. No. ab5694, ab7817; Abcam), CD163 (Cat. No. ab182422; Abcam), CD206 (Cat. No. A8301; Abclonal, Wuhan, China), Ki-67 (Cat. No. 12202; CST, Shanghai, China), Psmad3 (Cat. No. ab52903; Abcam), CXCR4 (Cat. No. ab124824; Abcam), FAP (Cat. No. ab28244; Abcam), TGF-β (Cat. No. ab92486; Abcam), and CXCL5 (Cat. No. ab9802; Abcam), recombinant anti-androgen receptor antibody [EPR1535(2)] (Cat. No. ab133273; Abcam), VENTANA anti-p63 (4A4) mouse monoclonal primary antibody (Roche), CXCR2 (Cat. No. ab225732; Abcam), Nkx3.1 (Cat. No. ab196020; Abcam).

### Flow cytometry

Cells were collected and washed twice with phosphate-buffered saline (PBS). The cells were then pre-incubated with human TrustainFcX^TM^ (Fc receptor blocking solution, Cat. No. 422301; BioLegend, San Diego, CA) for 10 min at room temperature. Cells were stained with antibody for 20 min on ice before analysis using a flow cytometer (FC500; Beckman, Shanghai, China). Flowjo V10 software was used for data analysis. All experiments were repeated three times. The antibodies used are as follows: PE anti-human CD163 (Cat. No. 333605; BioLegend), APC anti-human CD206 (Cat. No. 321109; BioLegend), and PE anti-human CXCR4 (Cat. No. 306505; BioLegend).

### Clinical dataset analysis

Clinical data of patients with PCa were obtained from The Cancer Genome Atlas (TCGA) and the Genotype-Tissue Expression (GTEx) databases. The gene expression profiling interactive analysis (GEPIA) dataset was downloaded from the website (http://gepia.cancer-pku.cn/). The GSE40272 and GSE6919 cohorts were downloaded from the GEO database. Data mining was performed as described in our previous [17].

### Cytokine arrays

Cytokine arrays (Proteome Profiler Human XL Cytokine Array Kit, Cat. No. ARY022B; R&D Systems) were used to test the changes in 105 cytokines and chemokines in CM obtained from TGF-β-induced CD14^+^ PBMCs treated with IgG, CXCL12, CAF CM, and CAF with neuCXCL12. First, primary CD14^+^ PBMCs were treated with 10 ng/mL TGF-β for 4 days to differentiate into M2 macrophages. PBMCs were seeded into six-well plates and cultured in a medium supplemented with IgG, CXCL12 (10 ng/mL), CM of CAF, and CM of CAF with neuCXCL12. After five days, the CMs were placed in an ordinary RPMI 1640 medium. After two days, the PBMC supernatants were collected and incubated overnight with the blocked membranes in separate dishes. Next, the membranes were washed and incubated with a diluted detection antibody cocktail for 1 h. Streptavidin-HRP was then used to incubate the membranes for 30 min and then washed away. Finally, each membrane was covered with Chemi Reagent Mix and exposed to X-ray film for the same time, and profiles of the mean spot pixel densities were analyzed using Image J software.

### Tissue microarrays (TMA) and scoring

We collected 131 samples and 15 paired tumors and lymph nodes from patients with PCa after radical prostatectomy in Fudan University Shanghai Cancer Center (FUSCC). Patient characteristics are listed in Supplemental Table S2. An approved and signed Institutional Review Board informed consent form was obtained from all patients. Written informed consent was obtained from all subjects before participation, and all protocols were approved by the Institutional Research Review Board at FUSCC. After IHC staining, the TMA chips of human PCa clinical samples were digitally scanned using a 3D HISTECH Pannoramic machine (Budapest, Hungary), and the whole field of each tissue spot was obtained for IHC evaluation. The expression levels of epithelial HIC1, stromal TGF-β, and CXCL5 were scored semi quantitatively based on staining intensity and distribution using the immunoreactive score (IRS). Briefly, immunoreactive score (IRS) = SI (staining intensity) × PP (percentage of positive cells). SI was assigned as follows: 0, negative; 1, weak; 2, moderate; and 3, strong. PP is defined as 0 = 0%; 1 = 0–25%; 2 = 25–50%; 3 = 50–75%; 4 = 75–100%. For categorization of the continuous IRS values into low and high, we chose a cutoff point for the measurements (range 0–12, cut point ≤4 versus >4). The mean count of positive CD206 cells was determined from three random fields of view (40×) and considered as the macrophage count. The primary antibodies used are as follows: HIC1 (Cat. No. bs-15485R; Bioss), CD206 (MRC1) (Cat. No. A8301; Abcam), CXCL5 (Cat. No. ab9802; Abcam). The ethics approval and consent to participate in this study were approved and consented by the ethics committee of FUSCC.

### Organoid culture

According to the protocol of prostate epithelial organoid culture [26], the mouse prostate was softly peeled and the seminal vesicles were removed. After washing in PBS with antibiotic, the pieces of the prostate were incubated in 2 mM EDTA/PBS for 10 min and further for 15 min at 4 °C. The crypt fractions were isolated and purified by successive centrifugation steps. 100 μL of a mixture of Matrigel (BD Biosciences) and complete growth medium (at a ratio of 2:1) and 20 μL of drops of crypt-containing Matrigel were added to pre-warmed wells in a 24-well plate. After polymerization, 600 μL of Advanced DMEM/F-12 (Invitrogen, Shanghai, China) containing growth factors (50 ng/mL EGF, 500 ng/ml R-spondin1, and 100 ng/ml Noggin; PeproTech, Rocky Hill, NJ;) was added and refreshed every two days.

### Animals and Galunisertib treatment

TRAMP-C1 was purchased from the American Type Culture Collection (ATCC^®^ CRL-2730). TRAMP-C1 was derived from the transgenic adenocarcinoma mouse prostate (TRAMP) model in C57BL/6 mice [27] which exhibited both histological and molecular features recapitulating many salient aspects of human prostate cancer. TRAMP-C1 cells were cultured in Dulbecco’s modified Eagle’s medium (DMEM) supplemented with 4 mM L-glutamine, 1.5 g/L sodium bicarbonate, 4.5 g/L glucose, 0.005 mg/ml bovine insulin, 10 nM dehydroisoandrosterone, 5% fetal bovine serum (FBS), 5% Nu-Serum IV, 1% penicillin, and 100 μg/mL streptomycin (https://www.atcc.org/products/crl-2730#required-products). TRAMP-C1cells were transfected with either a control vector (shCtrl) or a sh sequence directed against *Hic1* (shHic1). The C57BL/6J male mice received 5 × 10^5^ cells/mouse in the subcutaneous flank region at six weeks. When the solid tumor was palpable, the mice were treated with Galunisertib or CMC-Na through oral gavage twice per day (12 h intervals) for 14 consecutive days at a dosage of 200 mg/kg body weight. 2-Deoxy-2-[18F]-fluoro-D-glucose (^18^F-FDG) was injected into the tail vein for PET-CT analysis (0.1 mCi per mouse). We excluded one mouse in the shCtrl+Cmc-Na group. The missing mouse died on the 10th day after tumor inoculation without a clear reason. We speculate this may be due to repeated oral gavage-induced laryngeal edema. Given that the tumor curve is drawn in a continuous manner, we had to eliminate this mouse from the shCtrl+Cmc-Na group. Mice were sacrificed 24 h after the last administration of Galunisertib or CMC-Na by CO_2_ inhalation. A portion of the prostate or tumor tissue was placed in 10% neutral buffered formalin and embedded in paraffin. The other portion of the prostate or tumor tissue was snap-frozen and stored at −80 °C. All animal procedures were performed in accordance with the guidelines of the Institutional Animal Care and Use Committee of the Shanghai Jiao Tong University School of Medicine.

### Statistics

All data were analyzed using IBM SPSS Statistics 20 or GraphPad Prism 9.0. The significance of the differences between the control and experimental groups was evaluated using two-tailed Student’s *t*-tests. The migration curves recorded by the xCELLigence RTCA-DP system were evaluated using a two-way ANOVA. Kaplan–Meier curves for survival analyses were determined using the log-rank (Mantel–Cox) test. Spearman’s rank correlation coefficient analysis was performed to assess the relationship between epithelial HIC1, stromal CD206, TGF-β, ɑ-SMA, and CXCL5 in the PCa tissue microarrays. All values are expressed as the mean ± standard deviation, and significance was set at *P* < 0.05.

## Results

### Metastatic phenotype and prominent infiltration of M2 macrophages in *Pten*^−/−^*; Hic1*^−/−^ PCa model

**Fig. 1:**
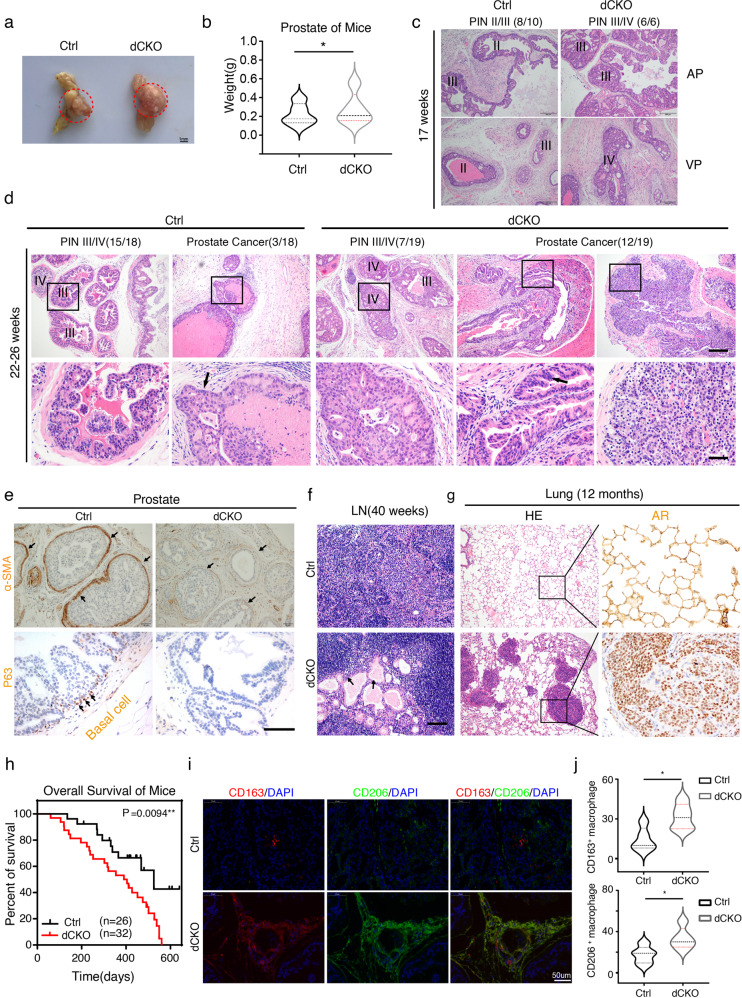
Prominent infiltration of M2 macrophages in *Hic1* knockout spontaneous PCa model. **a** Macroscopic image of prostate was dissected form from *Ctrl* and *dCKO* mice at 17 weeks old. Red dotted rounds represent dorsolateral prostates (DLPs) and ventral prostates (VPs). Scale bars: 1 mm. **b** Violin plot show prostate weight harvested from *Ctrl* and *dCKO* mice at 17 weeks old. (*n* = 5 each group, *P < 0.05, Mann–Whitney *U*-test). **c** H&E staining show prostatic intraepithelial neoplastic (PIN) lesions of anterior prostates (APs) and VPs from *Ctrl* and *dCKO* mice at 17 weeks old.8 out of 10 *Ctrl* mice show PINII or PINIII, while *dCKO* mice show 100% (6/6) PIN III or PINIV. Scale bars: 200 μm. **d** *Ctrl* and *dCKO* mice show different penetrance of PIN or prostate cancer at 22–26 weeks old. 12 of 19 *dCKO* mice progress into prostate cancer, while only 3 out of 18 *Ctrl* mice progress into prostate cancer. The majority of *Ctrl* mice pathological grade is high-grade PIN (PIN III or PIN IV). Box areas are magnified in the bottom panel. Black arrow indicates cancer invasion (*Ctrl*) or atypical nuclei (*dCKO*). **e** Representative images of α-SMA and P63 IHC staining of prostate at 17 weeks. Black arrows represent stromal fibroblasts (upper panel) or basal cells (lower panel). The basal cell layer is intact in *Ctrl* mice, while the integrity is disrupted in *dCKO* mice. Scale bars: 50 μm. **f** Scattered cancer cells formed gland-like structures (black arrows pointed) in the para-aortic lymph nodes at 40 weeks old in *dCKO* mice. Scale bars: 100 μm. **g** PCa cells spread to the lung at 12 months old in *dCKO* mice. Scale bars: 200 μm. **h** Kaplan–Meier analysis of *Ctrl* (*n* = 26) compared with *dCKO* (*n* = 32) mice (*P* = 0.0094, *P* value is obtained using the log-rank test). **i** Immunofluorescent staining shows infiltrated M2 macrophage in stroma on *Ctrl* and *dCKO* mice at 17 weeks old. **j** Violin plot shows quantitative analysis of the infiltrated M2 macrophages in stroma (*n* = 5 each group, *P < 0.05, Mann–Whitney *U*-test).

To investigate the *Hic1* in vivo functions, we generated double-conditional knockout mice by crossing *Hic1*^*flox/flox*^ and *Pten*^*flox/flox*^ mice with *PB-Cre* mice. The Cre-positive; *Pten*^*flox/flox*^*; Hic1*^*flox/flox*^ mice were classified as *dCKO*, while littermates Cre-positive; *Pten*^*flox/flox*^ mice were designated as the control group (here after *Ctrl*) (Supplemental Fig. S1a). Western blot (WB) assays indicated that Hic1 deletion occurred only in the prostate epithelium but not in the heart or liver (Supplemental Fig. S1b, c), which was confirmed again by HIC1 immunohistochemical (IHC) analysis (Supplemental Fig. S1d). The *dCKO* mice at 17 weeks of age exhibited larger dorsolateral prostate (DLP) and ventral prostate (VP) volume (Fig. 1a) and heavier weight of the whole prostate than that of the *Ctrl* mice (Fig. 1b). Meanwhile, knockout of *Hic1* accelerated tumor onset as shown in *dCKO* mice, which had higher pathological stage-high-grade prostatic intraepithelial neoplastic (PIN) lesions at 17 weeks of age (PIN III/IV vs. PIN II/III) and progressed into PRAD with a higher penetrance at 22–26 weeks old compared with *Ctrl* mice (12/19 vs. 3/18) (Fig. 1c, d). The *dCKO* mice displayed disruption of the basal cell layer as well as degradation of the basement membrane at 17 weeks of age (Fig. 1e). After 40 weeks of age, the *dCKO* mice exhibited obvious metastases where PCa cells spread to distant locations such as the lung, para-aortic lymph nodes (LNs), and adrenal glands, as verified by staining for androgen receptor (AR), NKX3.1, and/or Ki-67 (Fig. 1f, g, Supplemental Fig. S1f–i). These scattered cancer cells formed gland-like structures in the para-aortic lymph nodes (Fig. 1f, Supplemental Fig. S1f). Although both *Ctrl* and *dCKO* mice progressed to prostate cancer at 40 weeks of age (Supplemental Fig. S1g), Kaplan–Meier analysis demonstrated that *dCKO* mice exhibited shorter overall survival (OS) than the *Ctrl* group (*P* = 0.0094, Fig. 1h).

To determine the phenotype of TAMs in PCa, we used immunofluorescence staining to verify the status of infiltrated macrophages. Interestingly, both the *Ctrl* and *dCKO* mice were infiltrated with CD163^+^ CD206^+^ M2 macrophages. Compared with the *Ctrl* group, the number of tumor-infiltrating CD163^+^ (*P* = 0.03) and CD206^+^ (*P* = 0.03) macrophages was significantly higher in the *dCKO* group (Fig. 1i, j). Similar results were obtained by IHC analysis (Supplemental Fig. S1j, k). Taken together, these findings suggest that knockout of *Hic1* accelerated tumor onset and induced more M2 macrophage infiltration.

### HIC1 deleted-PCa cells induce migration and polarization of M2 macrophages

Given the increased infiltration of M2 macrophages in the prostate of *dCKO* mice, we further explored whether HIC1-deleted PCa cells had the ability to promote the process in vitro. To investigate this possibility, HIC1-deleted PC3 and C4–2B PCa cells (herein referred to as PC3 Ctrl/ PC3 sgHIC1 or C4-2B Ctrl/ C4-2B sgHIC1) were generated using the CRISPR-Cas9 system (Supplemental Fig. S2a). Next, we used the xCELLigence RTCA-DP System [28] to monitor the migration capacity of RAW264.7 cells induced by HIC1-deleted PC3 and C4-2B cells (Supplemental Fig. S2b). The curves of cell index within 48 h showed that these cells induced a higher motility capacity of the RAW264.7 macrophage cell line than the respective controls (Supplemental Fig. S2c). We isolated CD14^+^ human PBMCs from healthy volunteers, and the co-culture system was used to test the differentiation of macrophages (Supplemental Fig. S2d, e). Using flow cytometry, we found that CD163^+^CD206^+^ M2 macrophages at the 5^th^ day were significantly increased compared with the respective controls (Supplemental Fig. S2f). Similar effects were observed in RAW264.7 cell line (Supplemental Fig. S2g). Furthermore, we found that the supernatant derived from HIC1-deleted PCa cells could activate phosphorylation of the STAT3 signaling pathway in RAW264.7 cells (Supplemental Fig. S2h). In brief, these results suggest that HIC1-deleted PCa cells can directly induce the migration and transition of M2 macrophages in vitro.

### *TGFB1* is directly regulated by HIC1

**Fig. 2:**
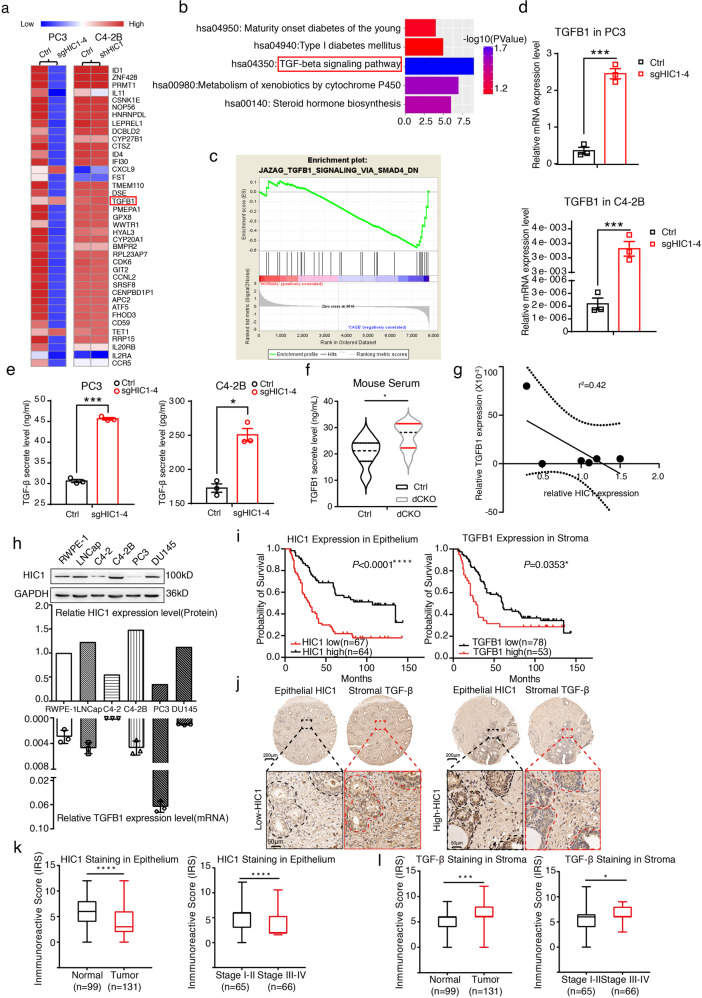
*TGFB1* is modulated by HIC1. **a** Hierarchical clustering heatmap shows a distinguishable mRNA expression profiling among groups. 39 DEGs were showed in here. Red box marks the most up-regulated gene TGFB1. **b** Top 5 enriched KEGG pathways among groups. Red box marks the most enriched pathway: the TGF-beta signaling pathway. **c** Gene set enrichment analysis (GSEA) enriched a set of differentially expressed genes named JAZAG_TGFB1_SIGNALING_VIA_SMAD4. **d** Real-time PCR analysis show *TGFB1* expression level in HIC1-deleted PC3 and C4-2B cells compared with the control. (***P < 0.001; two-tailed Student’s *t*-Test). **e** TGF-β secreted levels in culture supernatants of HIC1-deleted PC3 and C4-2B cells compared with the control. Supernatants were collected at 48 h and assayed via ELISA for TGF-β (*P < 0.05; ***P < 0.001. two-tailed Student’s *t*-Test). **f** ELISA analysis of TGF-β secretion levels in serum of *Ctrl* mice (*n* = 7) compared with *dCKO* (*n* = 10) mice. (*P < 0.05, Mann–Whitney *U*-test). **g** Pearson's correlation analysis showed that the*TGFB1* expression level correlates negatively with the HIC1 expression levels in cell lines which were commonly used in prostate cancer research (*R*^2^ = 0.421). **h** Upper panel: WB analysis demonstrated the expression of HIC1 in human PCa cell lines and human prostate epithelial cell line RWPE-1. Lower panel: Relative expression level of HIC1 and *TGFB1* in these cell lines. **i** Kaplan–Meier analysis of the probability of progression**-**free survival (PFS) in131 patients with PCa stratified by HIC1 and TGF-β expression, respectively. **j** Representative IHC images of TMA showing the corresponding expression of epithelial HIC1 and TGF-β in PCa tumor tissues. The black and red dashed lines indicate the epithelial area. Scale bar: 200 (upper panel) and 50 μm (lower panel). **k**, **l** Analysis of IRS score of HIC1 and TGF-β staining between normal and tumor, stage I–II and stage III–IV tissues. (*P* values were obtained using two-tailed Student’s *t* tests. The Whiskers connect the minimum and the maximum values to the Box; *P < 0.05, **P < 0.01, ***P < 0.001; ****P < 0.0001).

To explore potential downstream targets of HIC1, we next analyzed the genome-wide transcriptome profiles of PC3 Ctrl/PC3 sgHIC1 cells using Agilent Whole Human Genome Microarrays and combined analysis with our previous gene array data of C4-2B Ctrl/C4-2B shHIC1 cells (GSE78850). Among the differentially expressed 39 genes shown in Fig. 2a, TGFB1 was noted as the most representative gene, which encoded the critical TGF-β protein family. KEGG pathway analysis [29] showed the most enriched pathway associated with the increased mRNAs was the TGF-β signaling pathway (Fig. 2b, *P* = 0.018). GSEA [30] also showed similar results (Fig. 2c). Using RT-qPCR, we found that silencing *HIC1* in both cell lines greatly upregulated *TGFB1* (Fig. 2d). Meanwhile, deletion of *HIC1* induced higher levels of TGF-β secretion than the Ctrl (Fig. 2e). Moreover, TGF-β levels in serum were also higher in *dCKO* mice than in *Ctrl* group mice (Fig. 2f). Finally, we found that TGF-β expression level was inversely correlated with HIC1 in the cell lines commonly used in prostate cancer research (Fig. 2g, *R*^2^ = 0.421), especially in the three malignant cell lines, including C4-2B, PC3, and DU145 (Fig. 2h). To further confirm our findings, we examined the expression of epithelial HIC1 and stromal TGF-β using TMA in 131 patients with PCa (Supplemental Table S1). By Kaplan–Meier analysis, we confirmed that loss of HIC1 or higher TGFB1 expression levels correlated with lower progression-free survival (*P* < 0.0001 and *P* = 0.035, respectively) (Fig. 2i). In PCa samples, the cases with lower epithelial nuclear HIC1 levels often displayed higher stromal TGF-β expression than control and vice versa (Fig. 2j). In tumor tissue, IRS of epithelial HIC1 staining indicated that HIC1 expression was lower than that in normal tissue. In tumor tissues, HIC1 expression in stages III–IV was lower than that in stages I–II (Fig. 2k). Meanwhile, an opposite trend was observed for TGFB1 expression in these tumor tissues (Fig. 2l). We also analyzed the clinical association of HIC1 with PCa using public gene datasets of patients. Analysis of the TCGA+ GTEx and GSE40272 datasets indicated that HIC1 expression in tumor tissue was lower than that in adjacent normal prostate tissue for both (Supplemental Fig. S2i). Results showed that higher TGFB1 expression levels correlated with lower progression-free survival in the TCGA PRAD dataset as well as a lower OS ratio in TCGA Pan-Cancer (PANCAN) dataset by Kaplan–Meier analysis (Supplemental Fig. S2j, k), which was consistent with our previous findings (Fig. 2i–l). Altogether, these data suggest that TGF-β expression might be modulated by HIC1.

**Fig. 3:**
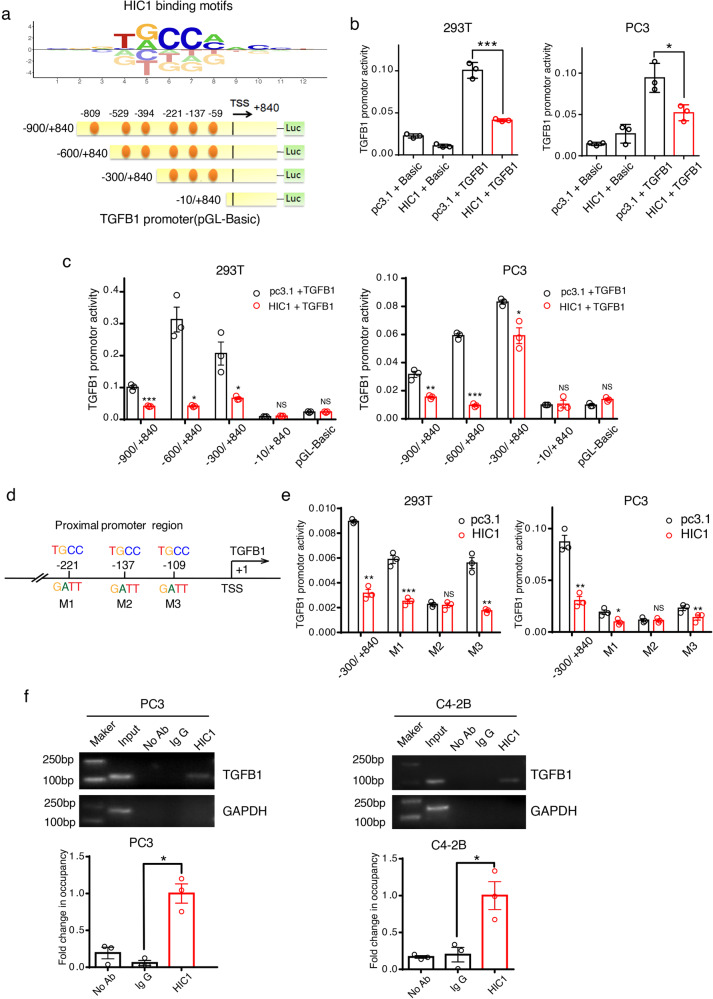
*TGFB1* is directly regulated by HIC1. **a** HIC1 binding motifs and putative HIC1-binding sites (yellow dots) are shown in *TGFB1* promoter region. Different lengths of the *TGFB1* promoter region were constructed for the dual-luciferase reporter assay. TSS: transcription start site. **b** *TGFB1* promoter activities were measured by luciferase reporter assays after transfection of the full-length construct (−900/+840) alone or together with HIC1 expression vectors in 293T and PC3 cells. pGL3-Basic is the control construct for promoter constructs; pc3.1 is the control vector for the HIC1 expression vector. **c** In 293T and PC3 cells, *TGFB1* promoter activities were measured by luciferase reporter assays after co-transfection with 100 ng of the HIC1 expression vector and each of the promoter constructs. The −10/+840 construct had no significant repressive effect, but other promoter constructs could all be markedly suppressed by HIC1 expression plasmid. **d** Mutations were identified and characterized in the promoter region of the*TGFB1*. Three potential HIC1 binding sites are marked with M1, M2 and M3 and mutated from TGCC to GATT. **e** In 293T and PC3 cells, reporter activity of the −300/+840 *TGFB1* promoter and corresponding three mutants (M1, M2, and M3) were measured after co-transfected with renilla luciferase and the HIC1 expression plasmid. Results show M2 mutated construct markedly neutralized the suppressive effect of HIC1 compared with the control. **f** ChIP-PCR/ qPCR analysis show HIC1are binding to the *TGFB1* promoter region in PC3 and C4-2B cells. (Mean ± SD; *P < 0.05, **P < 0.01, ***P < 0.001; *P* values were obtained using two-tailed Student’s *t-*Test; each experiment contained three biological replicates).

To identify whether *TGFB1* was a potential downstream target of HIC1, we further screened six putative HIC1-binding sites (https://motifcentral.org/publicfits; http://jaspar.genereg.net/analysis [31]) in the *TGFB1* promoter region (Fig. 3a). A series of TGFB1-truncated promoter/reporter fusion plasmids containing progressive deletions of the 5’ region of the gene from −900 to +840 were first constructed (Fig. 3a). These constructs were then transfected together with the pcDNA3.1-His or pcDNA3.1-HIC1 expression vectors into 293T and PC3 cells for luciferase reporter assays. With expectations, we found that the construct containing the full-length *TGFB1* promoter showed higher activity than the control construct. Moreover, transient transfection of the cells with HIC1 markedly inhibited *TGFB1* promoter activity (Fig. 3b) in a dose-dependent manner (Supplemental Fig. S3a). The suppressive effects of HIC1 on *TGFB1* promoter activity were observed in all the truncated constructs, including −900/+840, −600/+840, and −300/+840 (Fig. 3c). These results suggest that the HIC1-mediated repression region might be located within the −300 bp region of the *TGFB1* promoter, which contains three putative HIC1 binding sites (−221, −137, −109), named M1, M2, and M3 (Fig. 3d). We then mutated these sites (TGCC to GATT) to abolish the HIC1 binding function. Mutation of the M2 site abolished the repressive behavior of HIC1, suggesting that the M2 site in the *TGFB1* promoter was the key region in the HIC1-mediated suppression of *TGFB1* expression (Fig. 3e). Finally, the ChIP pulled-down DNA was amplified by ordinary PCR and RT-qPCR with primers designed based on the M2 site region of the *TGFB1* promoter. Our results suggest that TGFB1 promoter sequences were markedly enriched in the HIC1-immunoprecipitated PC3 and C4-2B chromatin but absent from the chromatin immunoprecipitated by the rabbit IgG control (Fig. 3f). In addition, we used the Cistrome Data Browser (http://cistrome.org/db/#/) [32] to verify whether HIC1 directly bound and negatively regulated the expression of *TGFB1*. In silico analysis showed that HIC1 accumulated largely in *TGFB1* promoter region in the HEK293 cell line (Supplemental Fig. S3b) [33]. In summary, these results demonstrate that HIC1 could directly repress *TGFB1* transcription.

### TGF-β promotes polarization of M2 macrophages and induces its CXCR4 expression via *c-Myc*

To explore whether TGF-β participated in the polarization of M2 macrophages, the xCELLigence RTCA-DP system was used to test the migration ability. As shown in Supplemental Fig. S4a, the migration of macrophages was markedly increased after TGF-β treatment for 48 h compared with the control, which was sustained until 72 h. Flow cytometry was performed to evaluate M2 marker expression after treatment with TGF-β. Taking IL-4, which could induce M2 polarization, as a positive control, we found that TGF-β treatment had a similar effect. M2 polarization was markedly suppressed by blocking the TGF-β signaling pathway with Galunisertib (Gal), an inhibitor of TGF-β receptor I (TGF-β RI) (Supplemental Fig. S4b). What’s more, we found that the mRNA expression level of TGFBR1 (encoding TGF-β receptor I), but not TGFBR2 (encoding TGF-β receptor II) in RAW264.7 cells was greatly upregulated, followed by a gradual increase in TGF-β treatment (Supplemental Fig. S4c). These results suggest that TGF-β promoted polarization of M2 macrophages by binding to TGF-β receptor I.

Unsurprisingly, we found that the Smad2/3 signaling pathway in RAW264.7 cells was markedly activated after TGF-β treatment for 20 min, which was greatly inhibited by Gal treatment (Supplemental Fig. S4d). Furthermore, CXCR4 was upregulated after TGF-β treatment, and this effect was greatly inhibited by Gal treatment (Supplemental Fig. S4e). Similarly, this effect was confirmed in the RAW264.7 cell line (Supplemental Fig. S4f). In addition, after TGF-β treatment at both 24 and 48 h, the increased CXCR4 expression was inhibited by Gal treatment, followed by a decrease in TGF-β receptor I expression, which suggests that TGF-β/TGF-β receptor I signaling is associated with CXCR4 (Supplemental Fig. S4g).

To further verify this effect, we used immunofluorescence staining to test the activation of the TGF-β signaling pathway and CXCR4 expression in the macrophages of our mouse model. As shown in Supplementary Fig. S4h, phosphorylation of smad3 (p-Smad3) and CXCR4 staining were both markedly increased in M2 macrophages derived from the *dCKO* mice compared with the Ctrl group, which was confirmed by quantitative analysis (Supplemental Fig. S4i). In conclusion, TGF-β signaling might play an important role in the polarization of M2 macrophages and the modulation of CXCR4 expression.

**Fig. 4:**
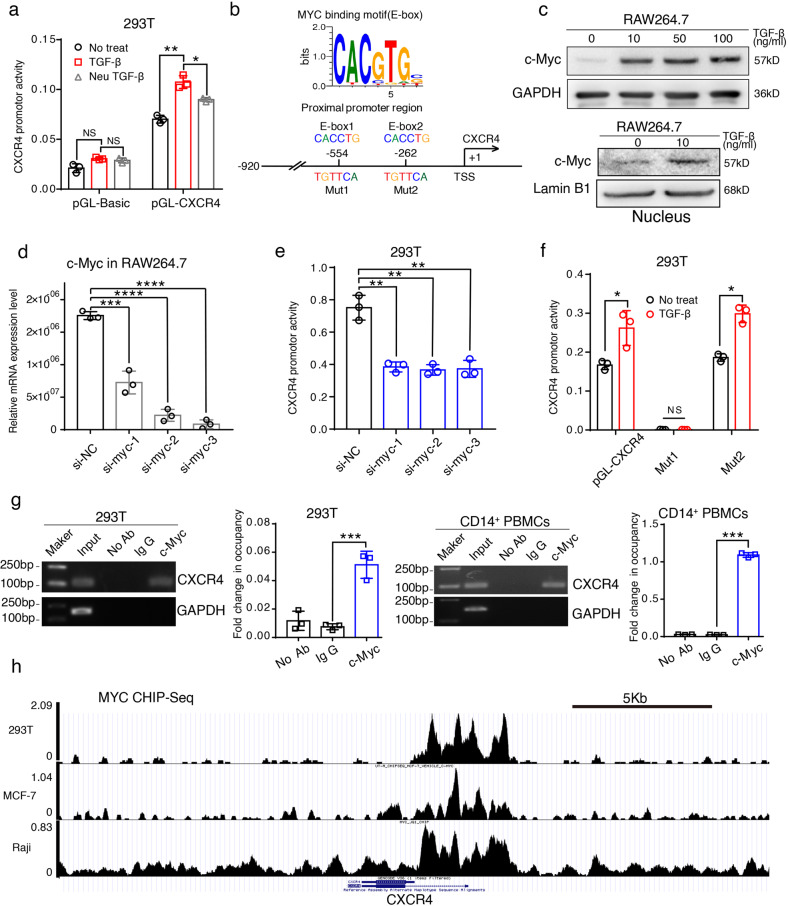
TGF-β promotes polarization of M2 macrophages and induces its CXCR4 expression *via c-Myc*. **a** CXCR4 expression is regulated by TGF-β and suppressed by TGF-β Neutralizing antibody. **b** Upper panel: MYC binding motif. Lower panel: bioinformatic analysis identified 2 putative E-box cis-elements (E-box1 and E-box2) in CXCR4 proximal promoter region. **c** TGF-β induced MYC expression in a dose-dependent manner and promote MYC translocation into the nucleus in RAW264.7 cells. **d** Utilize qPCR analysis to verify the expression level of *c-Myc* which is down-regulated using small interfering RNA (siRNA). **e** Silencing *c-Myc* inducing *CXCR4* promoter activity markedly decreased after co-transfecting *c-Myc* siRNAs in 293T cells. **f** Two potential MYC-binding sites are mutated from CACCTG to TGTTCA and marked with Mut1and Mut2 (see Fig. 5b lower panel). Then these mutant plasmids were co-transfected into 293T cells. Results show Mut1 mutated construct abolish the MYC-binding and activation effect at the *CXCR4* promoter region. **g** ChIP-PCR and ChIP-qPCR analysis demonstrated that *CXCR4* is a direct target of MYC. The pulled-down DNA was amplified by RT-qPCR and ordinary PCR with primers which were designed based on the Mut1 site of *CXCR4* promoter. **h** In silico analysis show c-MYC bound and regulated the expression of *CXCR4* in 293T, MCF-7, and Raji cancer cell lines.

Next, we explored the mechanism by which CXCR4 expression was induced in the TGF-β-mediated polarization of M2 macrophages. After transfecting the pGL vector containing the −920/+14 promoter of *CXCR4* into 293T cells, we measured the promoter activity using luciferase reporter assays. As shown in Fig. 4a, *CXCR4* promoter activity was stimulated by TGF-β and decreased by Neu TGF-β (TGF-β neutralizing antibody). To further investigate the transcriptional regulation of macrophage polarization, we utilized MotifMap (http://motifmap.ics.uci.edu/) [34] and PASTAA (http://trap.molgen.mpg.de/cgi-bin/pastaa.cgi) [35] to determine which transcription factors (TFs) directly participated in the regulation of *CXCR4*. In total, in silico analysis identified ~500 putative TFs/TF heterodimers that could regulate CXCR4 expressions, such as *HIF1A*, *ETS1*, *NF-kappa B*, and *GLI1/2* (Supplemental Table S2), which was consistent with a previous report [36]. Of these TFs, *c-MYC* draws more attention, which was increased 2–3 folds after TGF-β treatment [37, 38]. Two E-box cis-elements (5′-CACGTG-3′, as MYC-binding sites) were found in the CXCR4 proximal promoter region (Fig. 4b). Using WB assays, we found that c-MYC expression was increased in a dose-dependent manner after TGF-β treatment in RAW264.7 cells (Fig. 4c). Furthermore, TGF-β significantly enhanced the nuclear translocation of c-MYC (Fig. 4c). We knocked down *c-Myc* by siRNAs-mediated interference in RAW264.7, and detected *c-Myc* expression levels using qPCR (Fig. 4d). Notably, *CXCR4* promoter activity was markedly decreased after co-transfection of 293T cells with c-Myc siRNA compared with control siRNA (Fig. 4e). To assay the *c-Myc*-mediated activation effect on CXCR4, two putative E-box cis-elements in the *CXCR4* promoter were mutated from CACCTG to TGTTCA (Fig. 4b). As shown in Fig. 4f, *CXCR4* promoter activity was only markedly suppressed by the mutant construct of Mut1 site, which indicated that c-MYC preferentially bound to the sequence around the Mut1(−554) site of the *CXCR4* promoter region in 293T cells after treatment with TGF-β. To further verify whether *CXCR4* was a direct target of *c-Myc*, ChIP-PCR and ChIP-qPCR analyses were performed in 293T and CD14^+^ PBMCs using an antibody against c-MYC. CHIP results showed that c-MYC directly bound to the sequence of the E-box1 of *CXCR4* promoter in both 293T and CD14^+^ PBMCs, suggesting that c-MYC could upregulate the expression of *CXCR4* (Fig. 4g). Finally, using the Cistrome Data Browser, we also found that c-MYC bound and regulated the expression of *CXCR4* in several different human cancer cell lines (Fig. 4h) [39–41]. In brief, these results demonstrate that TGF-β regulates *CXCR4* transcription by inducing the expression of *c-Myc*.

### M2 macrophages secrete higher CXCL5 after crosstalk with CAFs which in turn promote PCa progression via EMT

**Fig. 5:**
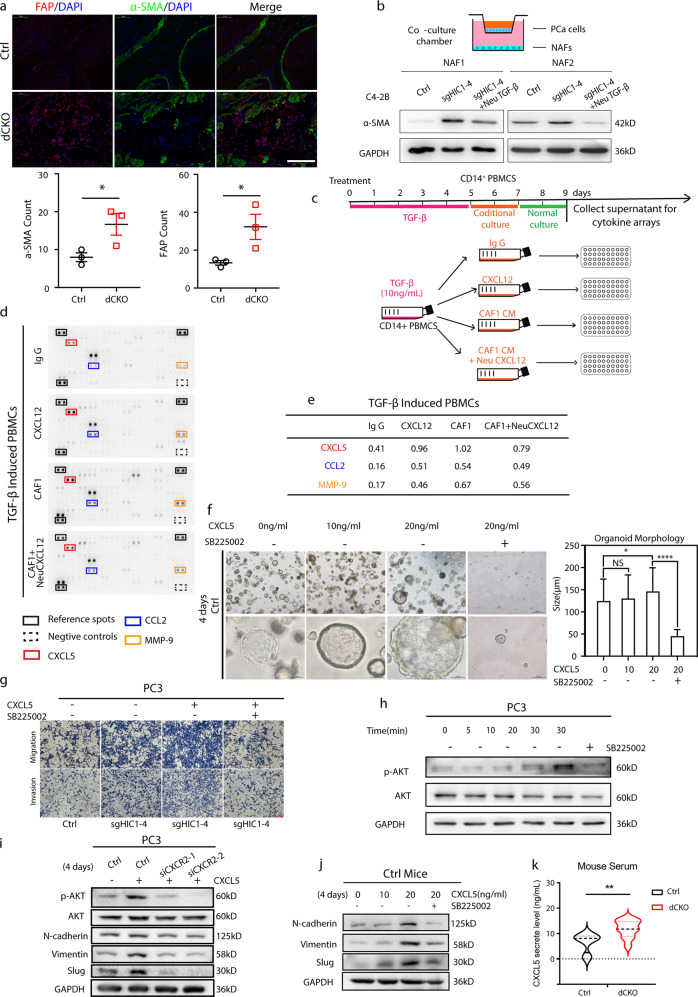
M2 macrophages secrete higher CXCL5 levels upon crosstalk with CAFs which in turn promote PCa progression via EMT. **a** Immunofluorescence staining of α-SMA and FAP in prostate stroma CAFs. The number of α-SMA and FAP positive cells in the prostate stroma were markedly increased in the *dCko* mice compared with the control mice (*P < 0.05. two-tailed Student’s *t*-Test). Scale bar: 50 μm. **b** Upper panel: schematic diagram of co-culture system. PCa cells were cultured in the upper compartment and the target cells (NFs) in the lower compartment of the well. Lower panel: WB analysis shows NFs are markedly activated when co-cultured with HIC1-deleted C4-2B cells, and these effects could greatly be abolished by using TGF-β neutralizing antibody. **c** Schematic diagram of the conditional culture system. CD14^+^ PBMCs culture with TGF-β for 5 days followed by 2 days of conditional culture with (1) IgG, (2) CXCL12, (3) CAF1 conditional medium (CM) or (4) CAF1CM with CXCL12 neutralizing antibody. Then transfer these cells into normal medium for 2 days. Discard the cells and collect the supernatant for cytokine arrays at the end of the culture. **d**, **e** Supernatant cytokine array results show CXCL5, CCL2 and MMP-9 were significantly increased in the CM of both CXCL12 activated-M2 macrophages and CAF1 activated-M2 macrophages compared with the corresponding control groups. **f** Treated with rhCXCL5 could promote prostate organoid growth, and this effect was markedly inhibited by SB225002. **g**–**i** The migration and invasive capabilities of PC3 cells were evaluated using a Transwell^®^ assay (24 wells; Matrigel gel; Corning, Inc.). Transwell experiments (**g**) and WB analysis (**h**, **i**) show treatment with rhCXCL5 enhanced the migration and invasive capabilities of PC3 sgHIC1 cells, and activation the AKT signaling pathway. These effects were partially abolished by SB225002. Knockdown CXCL5 receptor-CXCR2 in PC3 cells by siRNAs inhibited the EMT process and the AKT pathway (**i**). **j** The EMT phenotype of prostate organoids was also observed after treating with different concentrations of rhCXCL5 and markedly inhibited by SB225002. **k** ELISA analysis show *dCKO* mice have higher serum CXCL5 level than control group mice (*n* = 11 each group) (**P < 0.01, Mann–Whitney *U*-test).

The expression of α-SMA and FAP in the prostate stroma of *dCKO* mice was markedly increased compared with that in the Ctrl mice, which was confirmed by quantitative analysis (Fig. 5a). We isolated human primary fibroblasts from normal prostate glands (*n* = 6) or PCa tumor (*n* = 6) (marked as NF1-6/CAF1-6), respectively. The activation or inactivation status of fibroblasts was determined by WB analysis (Supplemental Fig. S5a). Next, a co-culture system was used to simulate NFs in vivo situations. As shown in Supplemental Fig. 5a, α-SMA expression was higher in CAFs than in NFs, indicating that CAFs were activated. We co-cultured representative two NFs (NF1 and NF2) with C4-2B Ctrl/C4-2B sgHIC1 or C4-2B sgHIC1 cells treated with TGF-β-neutralizing antibody (Fig. 5b). Two NFs were markedly activated when co-cultured with HIC1-deleted C4-2B cells and these effects could be greatly abolished by using a TGF-β neutralizing antibody (Fig. 5b). These findings indicate that TGF-β derived from HIC1-deleted PCa cells is involved in the activation of fibroblasts in vitro, which is consistent with the above effect in vivo.

CXCL12, the ligand of CXCR4, which is secreted from activated fibroblasts, was proven to stimulate cancer cell migration and promote cancer cell invasion through EMT transition [17, 25, 42], and is also elevated in CAF1 compared to NF1/NF2 (Supplemental Fig. S5b). By secreting CXCL12, CAFs attract conventional CD4^+^ T cell migration through its receptor-CXCR4 and promotes their differentiation into Foxp3^+^ regulatory T cells to create an immunosuppressive environment in human breast cancer [43]. Therefore, we hypothesized that these activated CAFs may also crosstalk with macrophages by binding to its cognate receptor CXCR4 to reshape the TME. To validate this hypothesis, we used cytokine arrays to detect the secreted soluble factors derived from CM of CXCL12-treated M2 macrophages, CM of CAF1-treated M2 macrophages, and CM of CAF1-treated M2 macrophages treated with CXCL12 neutralizing antibody (Fig. 5c, d). Figure 5d shows that three cytokines (CXCL5, CCL2, and MMP-9) were significantly increased in the CM of both CXCL12-treated M2 macrophages and CAF1-treated M2 macrophages compared with the corresponding control groups, which was confirmed by quantitative analysis (Fig. 5e). Among the three cytokines, CXCL5 exhibited the highest relative expression level. Consistent with cytokine arrays, ELISA analysis showed that CXCL5 levels were higher in the CM of CXCL12-treated M2 macrophages and CAF1 co-cultured M2 macrophages (Supplemental Fig. S5c). Next, to detect the effects of CXCL5 on PCa cells, we used an organoid model generated from *Ctrl* mice prostate treated with recombinant human CXCL5 (rhCXCL5) at different concentrations. First, we showed that the CXCL5 receptor, CXCR2, which was reported to be expressed in aggressive prostate cancer epithelial cells (GS ≥ 8 and CRPC patients) [44, 45], was also expressed in our spontaneous PRAD model as well as PC3 cells (Supplemental Fig. S5e). We found that CXCL5 greatly promoted the growth of organoids, which were then markedly inhibited by SB225002 (a potent and selective CXCR2 antagonist), and these results were confirmed by quantitative analysis (Fig. 5f). Meanwhile, using transwell experiments, we found that CXCL5 greatly enhanced the migration and invasive capabilities of PC3 sgHIC1 cells, and this effect was partially eliminated by SB225002 compared to the corresponding control (Fig. 5g), which was confirmed by quantitative analysis (Supplemental Fig. S5d). Mechanistically, treatment with rhCXCL5 markedly activated AKT signaling at different times, which was greatly inhibited by SB225002 (Fig. 5h). Furthermore, EMT phenotypes were observed in PC3 cells after treatment with rhCXCL5 for four days, followed by activation of AKT signaling. Knockdown of CXCR2 by siRNAs greatly inhibited these effects and the pathway (Fig. 5i, Supplemental Fig. S5f). Notably, the EMT phenotypes of organoids were also observed after treatment with different concentrations of rhCXCL5 and were markedly inhibited by SB225002 (Fig. 5j). These results suggest that the CXCL5/CXCR2 axis was responsible for PCa EMT through activation of AKT signaling. We also analyzed the clinical association of CXCR2 with PCa using public gene datasets of patients. Analysis of the GSE6919 dataset indicated that CXCR2 expression in metastatic tumor tissue was higher than that in primary tumor tissue (Supplemental Fig. 5g), which further confirmed our results. Moreover, we found *dCKO* mice had higher serum CXCL5 levels as well as stronger CXCR2 staining intensity in the prostate than control group mice (Fig. 5k, Supplemental Fig. S5e). In brief, these results demonstrated that upon crosstalk with CAFs, M2 macrophages secreted higher CXCL5 levels to enhance the malignant phenotypes of PCa by EMT.

### Galunisertib inhibits PCa growth and decrease M2 transition in vivo

**Fig. 6:**
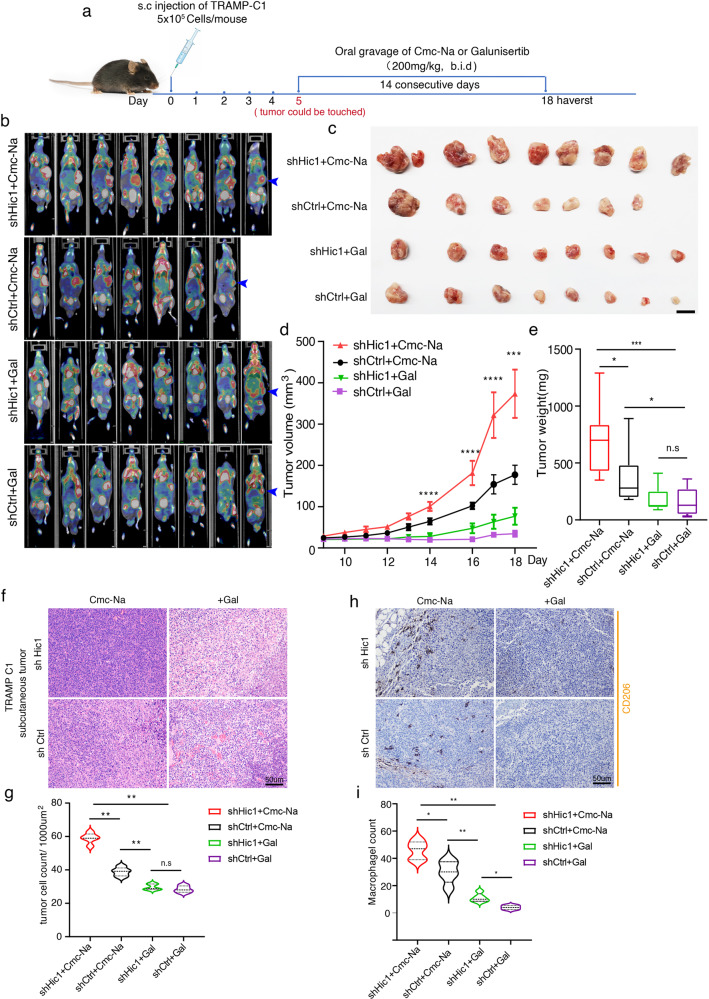
Targeting TGF-β pathway inhibits PCa development in vivo. **a** Flowchart to show the construction of syngeneic subcutaneous tumor models and in vivo drug testing. Galunisertib (Gal) or CMC-Na (control drug) are administered through oral gavage twice per day (12 h interval) for 14 consecutive days at a dosage of 200 mg/kg body weight when the solid tumor could be touched (~2–3 mm in diameter). **b** PET-CT results show TRAMP-C1 cells-derived tumor tissue exhibited ^18^F-FDG uptake in the subcutaneous flank region. TRAMP-C1-derived tumor tissue (the "light spots") detected on PET-CT is indicated by the blue arrowhead. **c** Macroscopic images of TRAMP-C1 cells-derived tumor tissue (*n* = 7–8 mice each group). Scale bars: 1 cm. **d**, **e** Quantitative analysis of tumor volume (**d**) and tumor weight (**e**) from TRAMP-C1 cells-derived subcutaneous tumor tissues. Tumor volume calculations were obtained using the formula *V* = (*W* × *W* × *L*)/2 for caliper measurements, where *V* is tumor volume, *W* is tumor width, *L* is tumor length. **f**, **g** Representative images of HE staining show after Gal treatment, the cell density of TRAMP-C1derived tumor tissue decreased compared with the corresponding control group (*n* = 5 each group, **P < 0.01, Mann–Whitney *U*-test). Scare bar: 50 μm. **h**, **i** Representative images of CD206 IHC staining show after Gal treatment, the number of M2 tumor-associated macrophages decreased compared with the corresponding control group (*n* = 5 each group, **P < 0.01, Mann–Whitney *U*-test). Scare bar: 50 μm.

Based on the above results, we believe the TGF-β signaling pathway promoted the activation of CAFs and the differentiation of macrophages into the M2 phenotype during PCa development, which in turn promoted PCa progression, suggesting that targeting TGF-β signaling may offer a therapeutic strategy for the disease. Galunisertib (LY2157299) is a small molecule inhibitor of TGF-β receptor I kinase that has been investigated in animal models and patients with cancer [46]. To test this hypothesis, the murine prostate cancer cell line TRAMP-C1 (ATCC CRL-2730) pre-transfected with either a control vector (shCtrl) or a sh sequence directed against *Hic1* (shHic1) was injected into the subcutaneous flank region of C57BL/6J mice. On day 5 after inoculation, the solid tumor was palpable (~2–3 mm in diameter). These mice were then treated with Gal or CMC-Na (control drug) through oral gavage twice per day (12 h intervals) for 14 consecutive days at a dosage of 200 mg/kg body weight (Fig. 6a). PET-CT results showed that in mice without Gal treatment, both TRAMP-C1(shHic1) and TRAMP-C1(shCtrl) cell-derived tumor tissues exhibited larger tumor volumes and higher ^18^F-FDG uptake in the subcutaneous flank region, which was further confirmed by quantitative analysis (Fig. 6b–e). However, when treated with Gal, both TRAMP-C1 (shHic1) and TRAMP-C1 (shCtrl) cell-derived tumor tissues exhibited significant tumor growth inhibition and lower tumor weight, indicating that targeting TGF-β signaling was able to prevent tumor growth (Fig. 6c–e).

In addition, HE staining showed that tumor cell density decreased after Gal treatment (Fig. 6f, g), while M2 macrophages were barely observed in the tumor region when treated with Gal (Fig. 6h, i). This confirmed that Gal treatment inhibited the TGF-β pathway activity. Taken together, these findings indicate that blocking the TGF-β pathway could inhibit the development of PCa by decreasing M2 macrophage infiltration and polarization.

### The Cancer cell-CAF-Macrophage self-reinforcing paracrine loop is associated with the progression of PCa patients

**Fig. 7:**
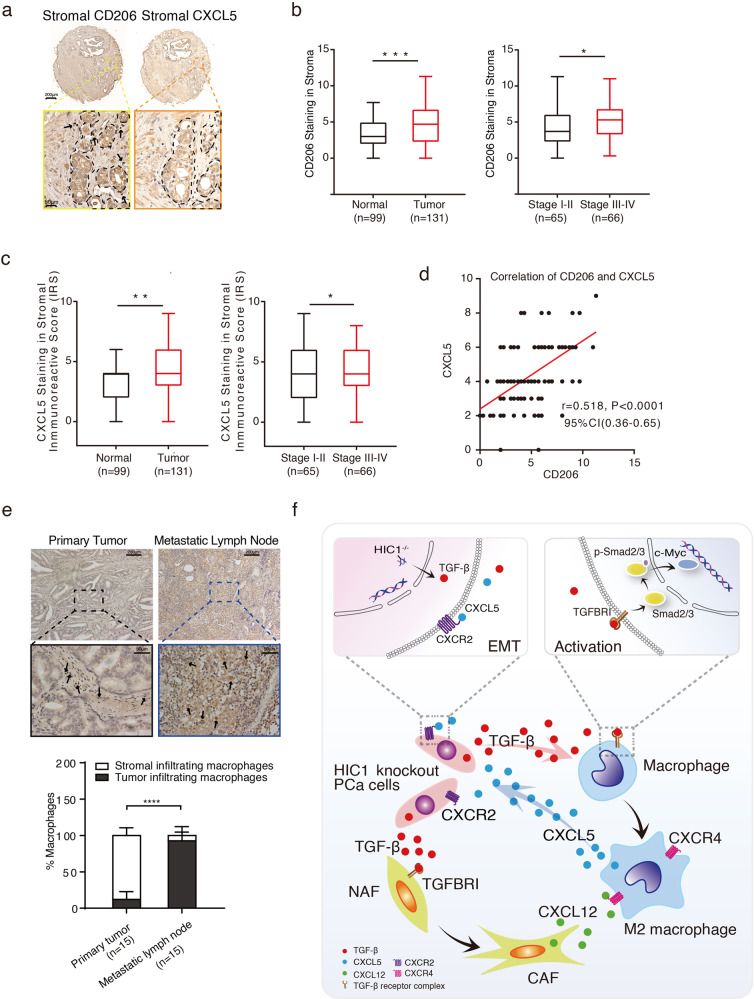
The HIC1-TGFB1-CXCL5 loop is associated with disease progression in patients with PCa. **a** Representative IHC images of TMA showing the corresponding expression of stromal CD206 and CXCL5 in PCa tumor tissues. Scale bar: 200 (upper panel) and 50 μm (lower panel). **b** Analysis of a number of CD206 staining cells in stroma between normal and tumor, stage I–II and stage III–IV tissues. **c** Analysis of CXCL5 staining in stroma between normal and tumor, stage I–II and stage III–IV tissues (*P* values were obtained using two-tailed Student’s *t* tests. The Whiskers connect the minimum and the maximum values to the Box; *P < 0.05, **P < 0.01, ***P < 0.001; ****P < 0.0001). **d** The correlation between CD206^+^ cells and CXCL5 expression levels in stroma. Spearman *r* = 0.541. The correlation was calculated by Spearman correlation analysis. **e** IHC staining of stromal CD206 in primary tumor and metastatic lymph nodes from the same patient. Black arrows represent M2 macrophages. Lower panel: quantitative analysis of infiltrating macrophages in primary tumor compared with infiltrating macrophages in metastatic LNs. *P* values were obtained using two-way ANOVA. Scale bar: 200 (upper panel) and 50 μm (lower panel). **f** Schematic model shows how HIC1-mediated crosstalk between cancer cells, macrophages, and fibroblasts promotes in the process of PCa progression. Conditional deletion of *Hic1* and *Pten* in mouse prostate epithelium contributes to the malignant phenotype of PCa. HIC1-induced higher TGF-β secretion in TME promotes M2 macrophage activation and up-regulates CXCR4 expression by inducing c-Myc pathway. Meanwhile, TGF-β activates NFs into CAFs to secrete higher CXCL12, which binds to CXCR4 on M2 macrophages. Upon the interaction with CAFs, M2 macrophages in turn enhance the malignant phenotypes of PCa through induction of EMT by activating the CXCL5/CXCR2 chemokine axis.

Our results showed that PCa cells secreted TGF-β to target macrophages and fibroblasts, and activated macrophages secreted CXCL5, which in turn promotes EMT transition of PCa cells *via* CXCR2. To further confirm our findings, we examined the expression of stromal CD206 and stromal CXCL5 using TMA in 131 patients with PCa (Supplemental Table S1) (Fig. 7a). Quantitative analysis of stromal CD206 staining showed that more M2 macrophages were present in tumor tissues than in normal tissues. In this case, IRS analysis of stromal CXCL5 staining indicates that CXCL5 expression levels in tumor tissues were higher than those in normal tissues (Fig. 7b). Moreover, the higher the malignancy of PCa, the more CD206 staining infiltration was observed (Fig. 7b, right panel). In this case, IRS analysis of stromal CXCL5 staining indicates that CXCL5 expression levels in tumor tissues were higher than those in normal tissues (Fig. 7c). Moreover, CXCL5 expression levels between stages I and II and III–IV showed the same trend as CD206 (Fig. 7c). Furthermore, we found a positive correlation between CD206 and CXCL5 expression levels in the stroma (Fig. 7d, *r* = 0.518). To explore whether infiltration of M2 macrophages was responsible for metastasis of PCa, CD206 staining was performed in 15 primaries and paired metastatic LNs (Supplemental Table S1). The percentage of CD206 staining indicated that M2 macrophages assembled in the stroma of the primary tumor, while infiltration of M2 macrophages was observed in the tumor sites of metastatic LNs (Fig. 7e). Taken together, these results suggest that the HIC1-TGFB1-CXCL5-CXCR2 loop was associated with the progression of PCa in patients (Fig. 7f).

## Discussion

In the process of cancer development, the loss or attenuation of tumor suppressor gene function is often caused by mutations or aberrant methylation of gene promoters. DNA methylation in the promoter region of tumor suppressor genes often lead to gene silencing and carcinogenesis [47]. HIC1 is a tumor suppressor gene located at 17p13.3, which resides completely within a CpG island that is frequently hypermethylated in human tumors, including medulloblastoma, prostate, and lung cancer [48–50]. Our previous work showed that the HIC1 promoter was highly methylated in human PCa cell lines and tissues, resulting in the silencing of HIC1 expression [25, 48]. Mechanically, loss of HIC1 expression may be involved in the metastasis and invasion of PCa by triggering EMT transition [25]. However, the role of HIC1 in modulating the TME in PCa remains largely unknown.

Notably, we confirmed that HIC1 suppresses the epithelium secretion of TGF-β, which is an important functional protein in tumor development and malignancy [51]. TGF-β was reported to play a role in modulating biological processes such as cell invasion, immune regulation, microenvironment modification, and M2 macrophage polarization [51]. Zhang et al. reported that TGF-β modulated the expression of *SNAIL* (a transcriptional repressor controlling EMT) to change the phenotype of M1 and M2-like differentiation of macrophages [52]. Tauriello showed that increased TGF-β in the TME represented a primary mechanism of immune evasion that promoted T-cell exclusion and blocked the acquisition of the TH1-effector phenotype [53]. In the present study, we found that TGF-β enhanced the crosstalk between tumor cells, M2 macrophages, and CAFs to trigger a series of biological processes. Smad2/3 signaling pathway is the downstream pathway of TGF-β/TGF-β receptor I [54]. TGF-β promoted M2 macrophage transition by activating STAT3 signaling and CXCR4 expression. STAT3 signaling is well known as a key signaling pathway related to the polarization of M2 macrophages [55].

CXCR4 belongs to the large superfamily of G protein-coupled receptors, which have been reported to participate in several important biological processes, such as organogenesis, hematopoiesis, and vascularization [56]. Recently, CXCR4 upregulation has been shown to be highly associated with TAM recruitment and M2 polarization [57, 58]. Arwert et al. found that breast cancer cells secreted TGF-β to upregulate *CXCR4* in monocytes, while perivascular fibroblasts secreted CXCL12 to attract these monocytes into tumor beds to promote cancer cell invasion [57]. However, the mechanism by which TGF-β regulates *CXCR4* expression remains unknown. Through bioinformatic analysis, we found that *c-Myc* had specific binding motifs in the *CXCR4* promoter region. In line with the ChIP experiments, we found that *c-Myc* directly bound to the E-box DNA consensus sequence and regulated the transcription of *CXCR4*. Our results demonstrate that TGF-β promoted the transition of M2 macrophages and induced CXCR4 expression via *c-Myc*.

We first revealed that TGF-β could target *c-Myc* to upregulate CXCR4 expression, which shed light on the long mystery in this field. However, we still do not fill the gap that how TGF-β upregulate the *c-Myc*. Previous studies showed that *c-Myc* is a downstream target of the Smad pathway [59–61]. However, the traditional view believed that canonical TGF-β signaling represses *c-Myc* transcription [62]. While the non-canonical TGF-β signaling effector, BMP, is able to induce interaction of Smad1 with TCF4 and β-catenin to stimulate *c-Myc* transcription [62], so we speculated that TGF-β may enhance the *c-Myc* expression by non-canonical TGF-β/BMP signaling. Furthermore, we also illustrated a self-reinforcing feedback loop in the pathophysiological process of PCa, which was realized by that epithelium/TGF-β-fibroblasts/CXCL12-M2 macrophages/CXCL5 trajectory, and finally in turn to enhance the malignant phenotype of PCa epithelial cells by EMT. In nasopharyngeal carcinoma, CXCL5 contributes to EMT by activating ERK/GSK-3β/Snail signaling [63]. CXCL5 derived from TAMs in gastric cancer promotes metastasis by activating the CXCR2/STAT3 feed-forward loop [64]. Notably, CXCL5 was reported to promote tumor cell migration and invasion in PCa, especially in relation to bone metastasis [65]. CXCL5 serum levels were higher in patients with metastatic PCa than in patients with localized PCa [66]. Here, we show that CXCL5 derived from M2 macrophages stimulates the AKT signaling pathway and contributes to the progression of PCa through EMT.

Recently, strong evidence showed that high TGF-β secretion enhanced Th17 subsets in bone metastases of patients who were diagnosed with metastatic castration-resistant prostate cancer (mCRPC), suggesting that the TGF-β blockade with immune checkpoint therapy increased Th1 subsets and therefore, contributed to CRPC treatment [67]. Moreover, a recent study indicated that enzalutamide-exposed adenocarcinoma cells upregulated expression programs associated with EMT and TGF-β signaling [68], which suggests that targeting TGF-β signaling may be beneficial for precise PCa treatment. In this study, we used the drug Gal, a TGF-β receptor antagonist, markedly inhibited the progression of PCa in *Pten* and *Hic1* double conditional knockout mice. Gal was reported to have antitumor activity in tumor-bearing animal models, such as gastric cancer, lung cancer, and hepatocellular carcinoma [52, 69, 70]. Moreover, Gal was investigated either as monotherapy or in combination with standard anti-tumor regimens in patients with cancer, including glioblastoma, pancreatic cancer, and hepatocellular carcinoma. However, the cardiac toxicities and drug resistance of Galunisertib are the major concerns and challenges for its clinical application [71]. Developing a new TGF-β blockade with fewer side effects is promising. Our findings indicate that targeting TGF-β signaling using small molecule inhibitors may provide a strategy for advanced PCa therapy.

In PCa, the infiltration of TAMs and CAFs has important clinical significance and is closely related to the clinical T stage and prognosis of patients. Several relevant clinical indicators (such as serum PSA level, Gleason score, and T stage) were positively correlated with the infiltration of TAMs and CAFs. At the same time, patients with less infiltration of TAMs and CAFs had significantly higher recurrence-free survival (RFS) and better hormonal therapy outcomes than patients with more infiltration [72]. Therefore, controlling the infiltration of TAMs and CAFs can achieve better therapeutic effects. For example, many studies reported that inhibiting the GPR30 receptor on the cell surface of CAFs can inhibit the level of CXCL12 secreted by CAFs, reduce the recruitment of macrophages and M2-type polarization, thereby impairing the ability of prostate cancer cells to metastasize and invade [73]. In this study, we examined the expression of stromal CD206 and stromal CXCL5 in 131 patients and found a positive correlation between CD206 and CXCL5 expression levels in the stroma. In 15 primaries and paired metastatic LNs, the high level of CD206 staining in LNs indicated that M2 macrophages infiltration may be involved in the metastasis of PCa. The sample size needs to be improved in our further study.

In summary, we showed that the TME was shaped by TGF-β in HIC1-deficient PCa. The TGF-β receptor I inhibitor Galunisertib blocked the HIC1-TGF-β-CXCL5-CXCR2 signaling circuit, which generates robust therapeutic targets for PCa.

## Supplementary information

Checklist

Supplemental Figure S1

Supplemental Figure S2

Supplemental Figure S3

Supplemental Figure S4

Supplemental Figure S5

Original Data File-western blot

Supplemental table S1-Patient characteristics

Supplemental table S2

Supplemental Figure legend and materials

## Data Availability

All data are included in this article and its supplementary materials or available upon request to the corresponding author. The gene expression matrices files generated in this study were deposited in the NCBI Gene Expression Omnibus (GEO) database (GSE141642).
