# Supplementary figures and images for: Targeting HIC1/TGF-β axis-shaped prostate cancer microenvironment restrains its progression
Source: Cell Death Dis. 2022 Jul 19;13(7):624. doi: 10.1038/s41419-022-05086-z (PMC9296670; doi:10.1038/s41419-022-05086-z)

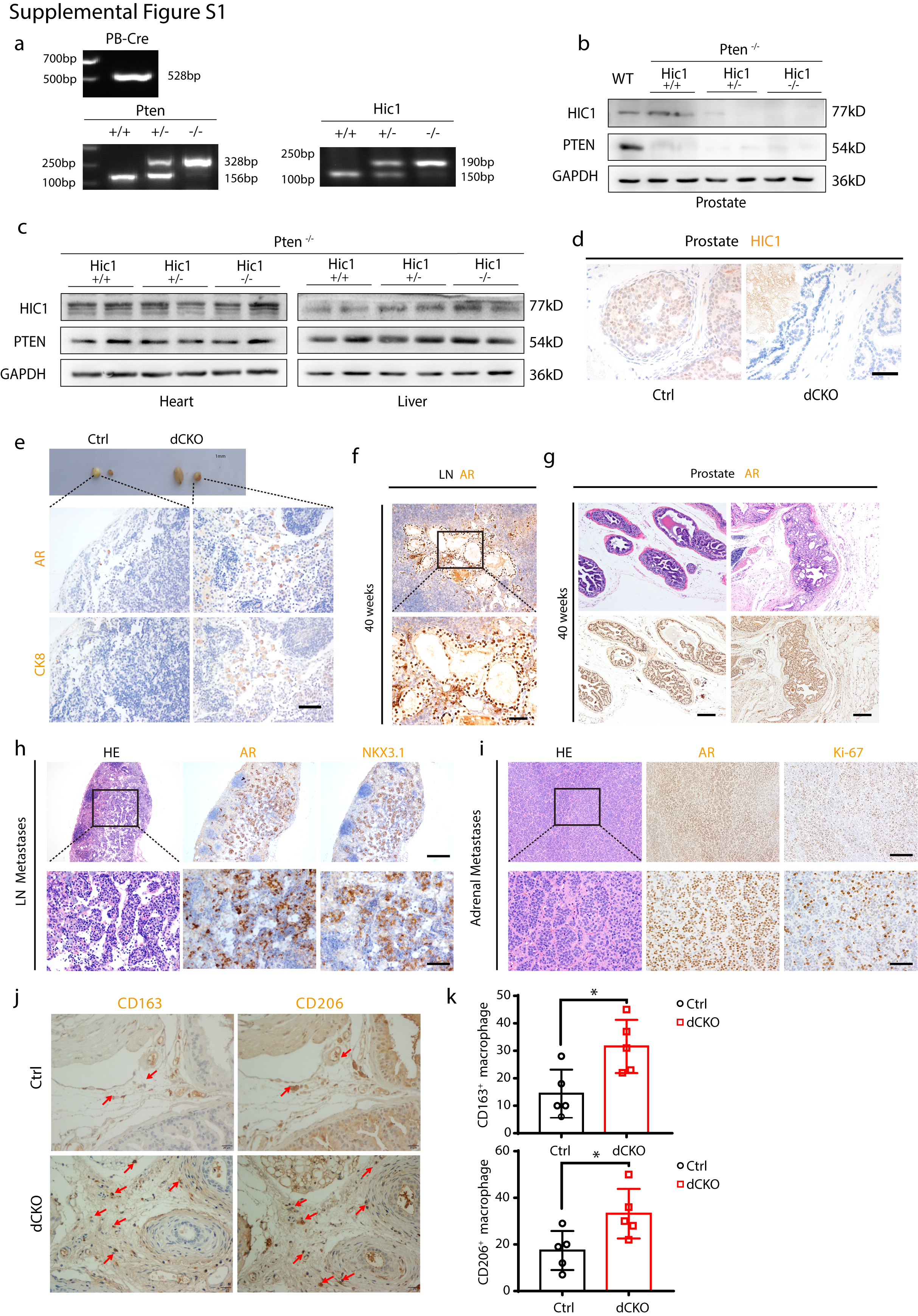

Supplement: Supplementary file 2 — Supplemental Figure S1 [file 41419_2022_5086_MOESM2_ESM.tif]

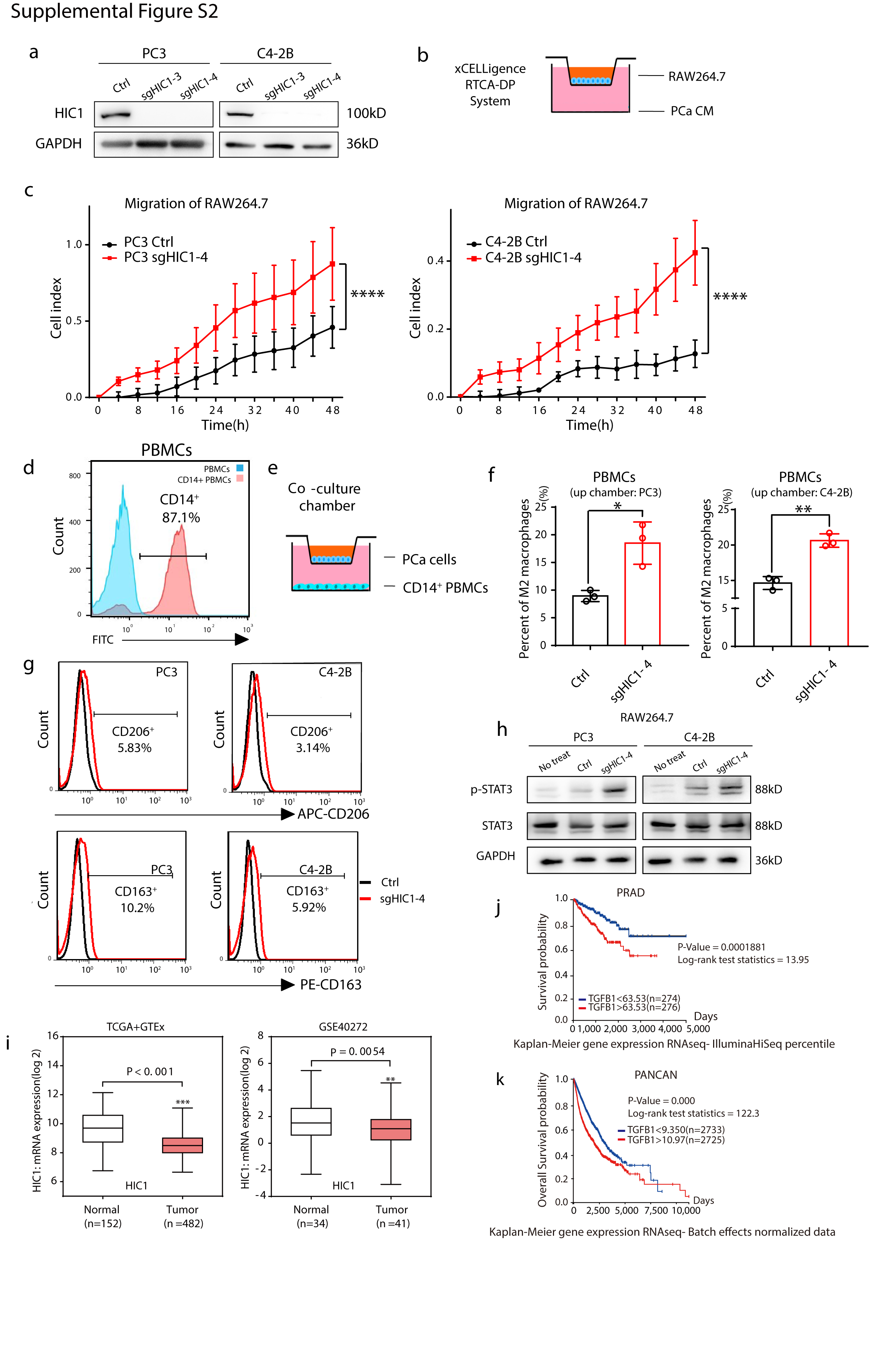

Supplement: Supplementary file 3 — Supplemental Figure S2 [file 41419_2022_5086_MOESM3_ESM.tif]

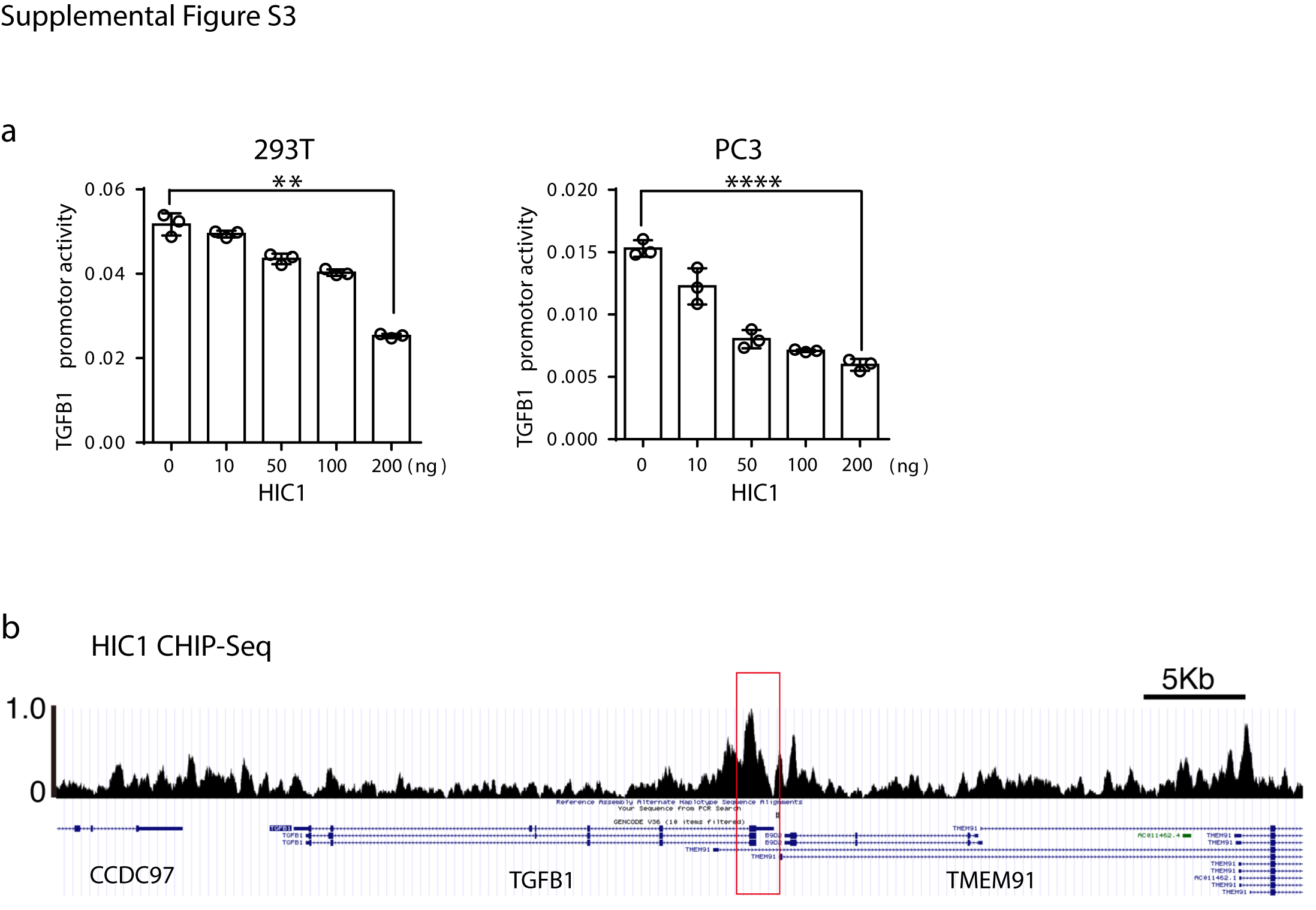

Supplement: Supplementary file 4 — Supplemental Figure S3 [file 41419_2022_5086_MOESM4_ESM.tif]

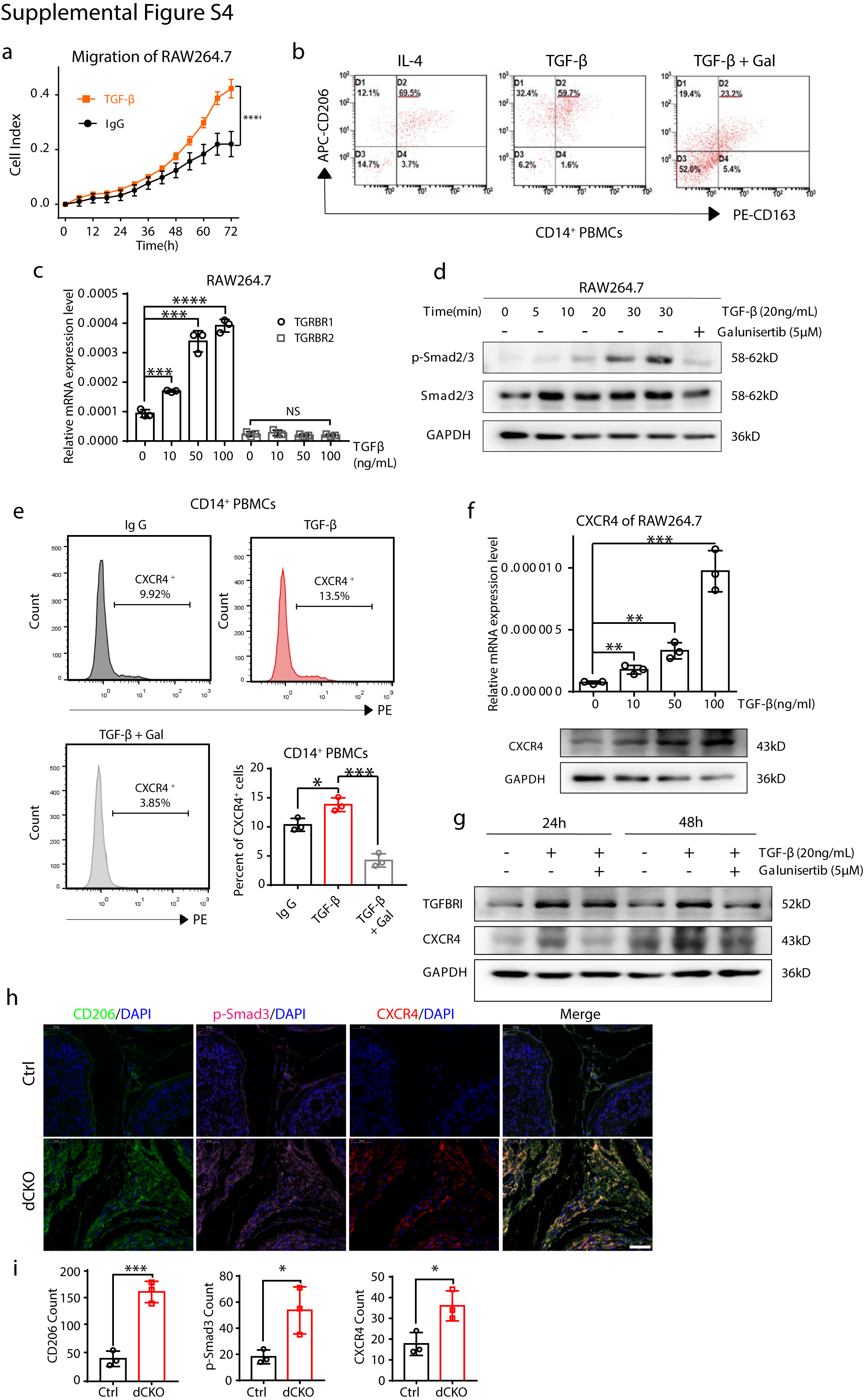

Supplement: Supplementary file 5 — Supplemental Figure S4 [file 41419_2022_5086_MOESM5_ESM.tif]

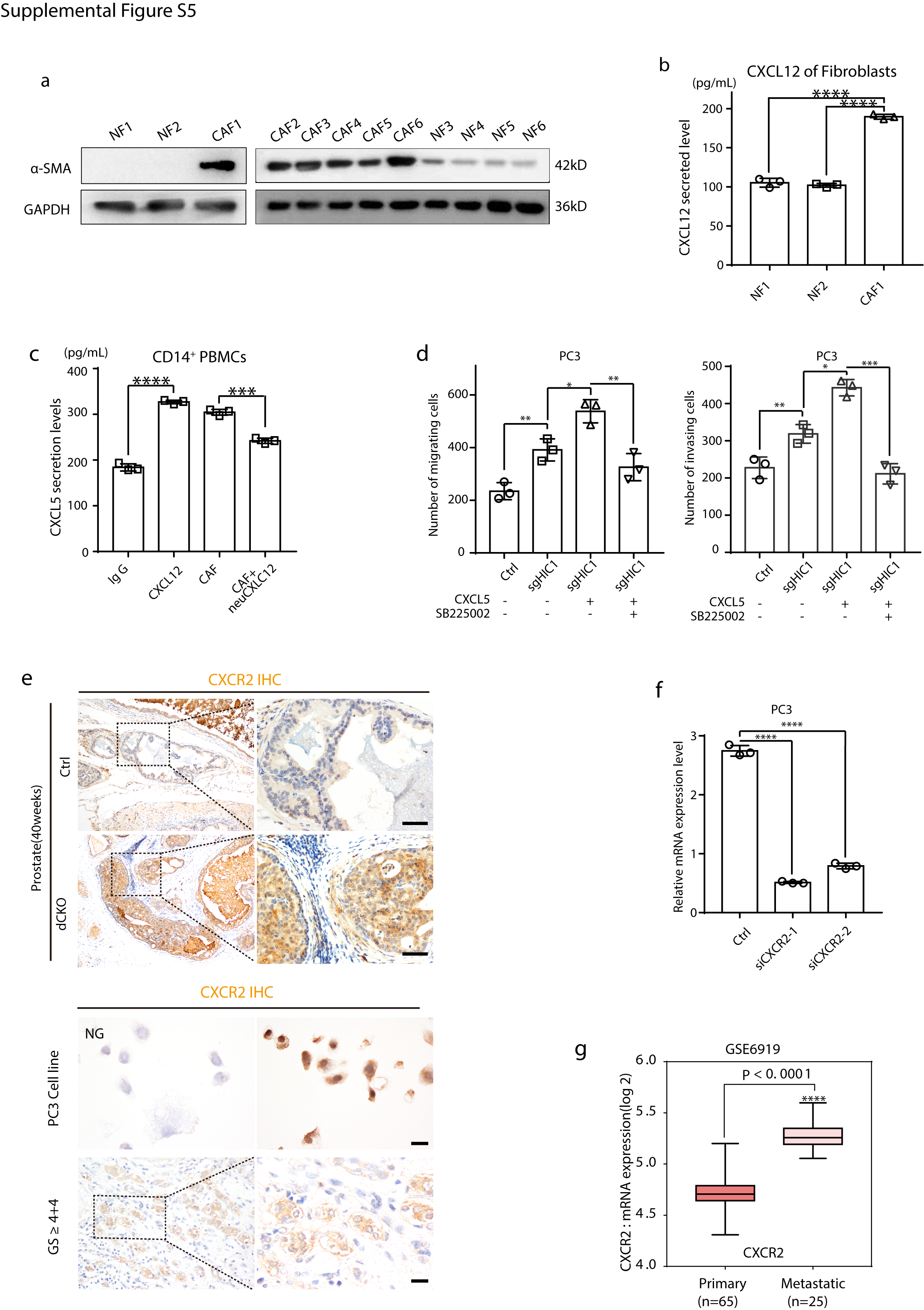

Supplement: Supplementary file 6 — Supplemental Figure S5 [file 41419_2022_5086_MOESM6_ESM.tif]
