# Supplementary material for: Targeting HIC1/TGF-β axis-shaped prostate cancer microenvironment restrains its progression
Source: Cell Death Dis. 2022 Jul 19;13(7):624. doi: 10.1038/s41419-022-05086-z (PMC9296670; doi:10.1038/s41419-022-05086-z)

Figure 2h.

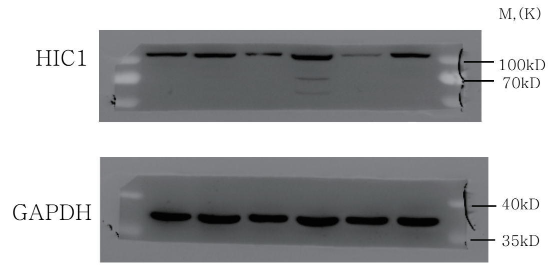

Figure 4c.

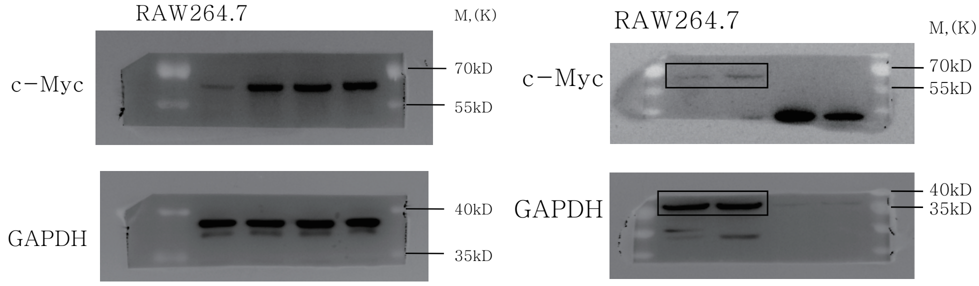

Figure 5b.

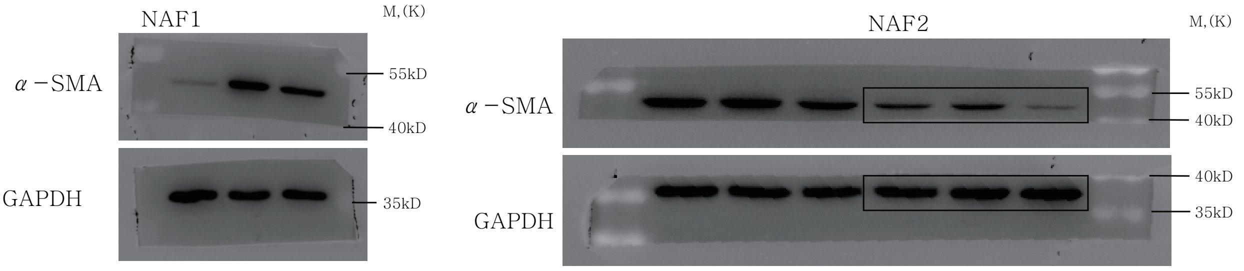

Figure 5h.

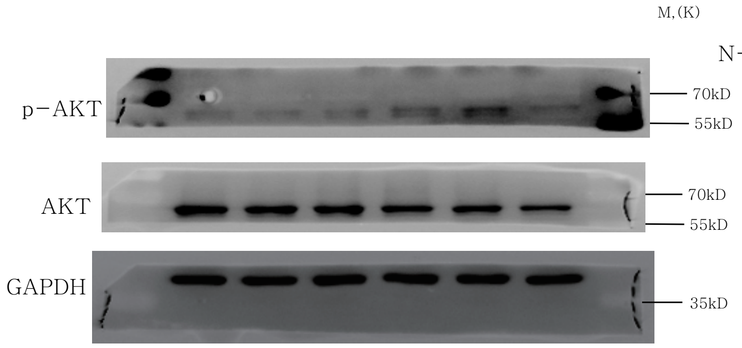

Figure 5i.

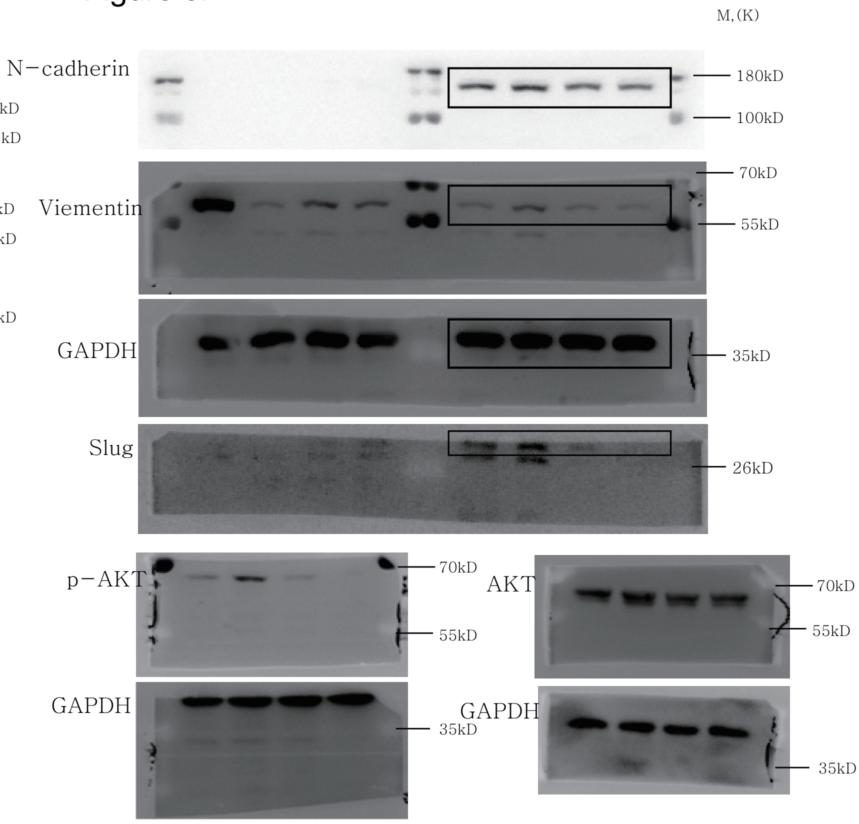

Figure 5j.

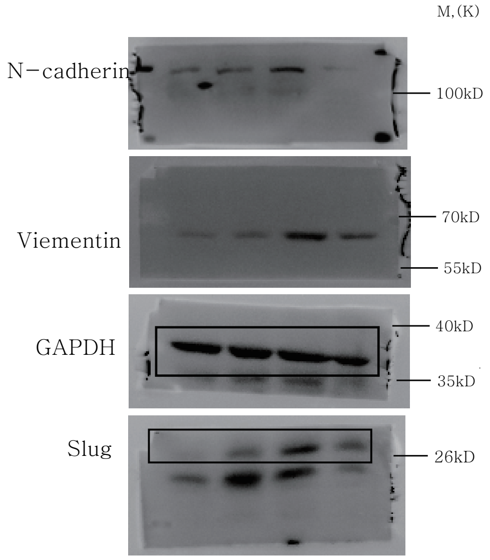

Supplemental Figure S1b.

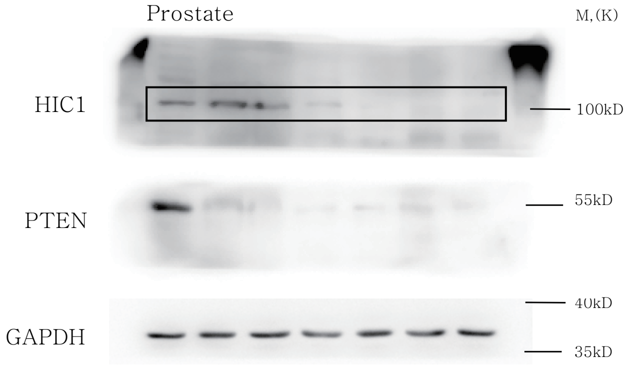

Supplemental Figure S1c.

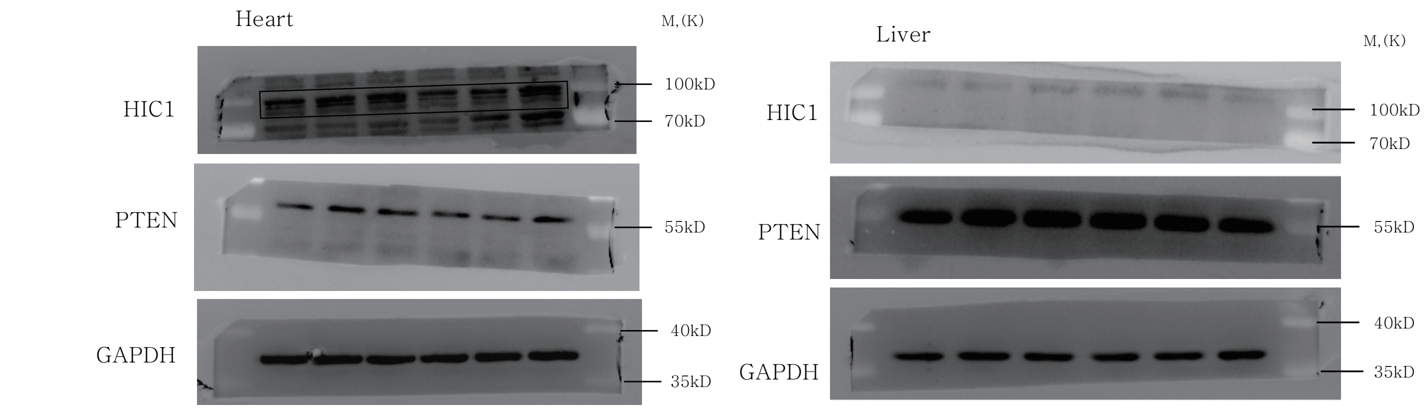

Supplemental Figure S2a.

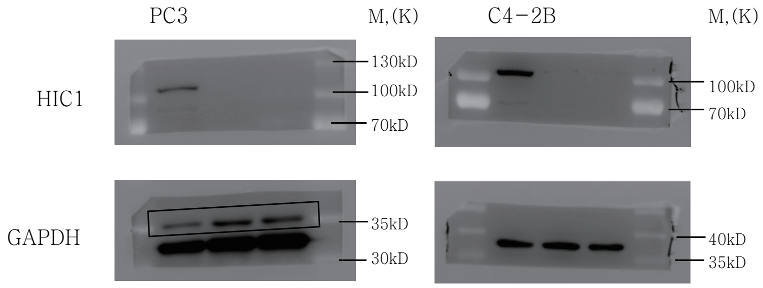

Supplemental Figure S2h.

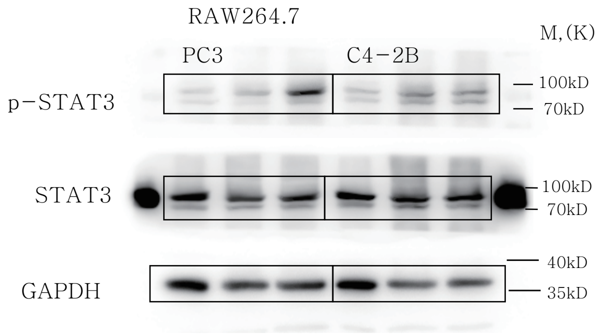

Supplemental Figure S4d.

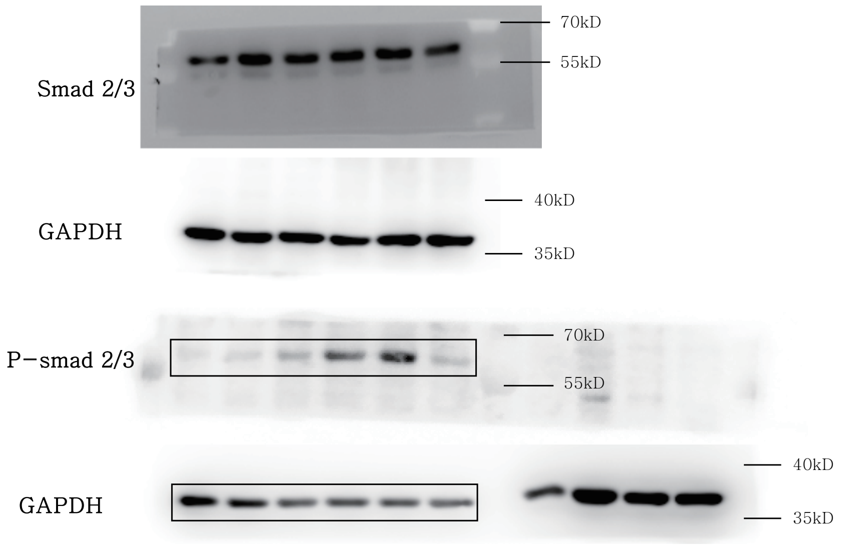

Supplemental Figure S4f.

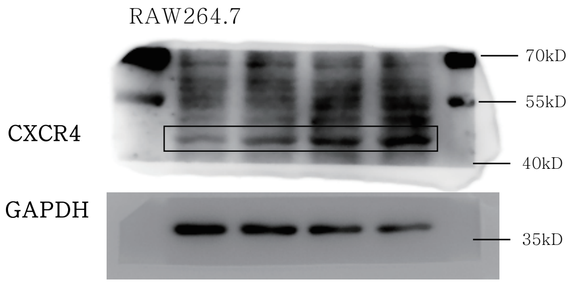

Supplemental Figure S5a.

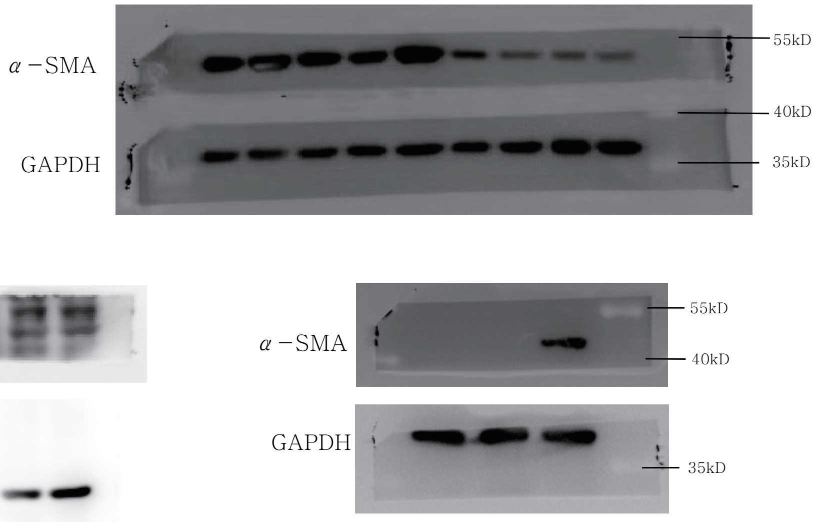

Supplemental Figure S4g.

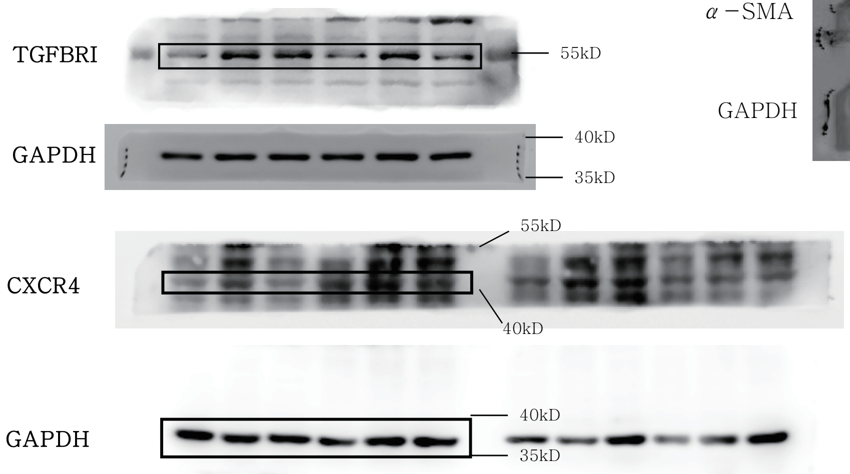

Supplement: Supplementary file 7 — Original Data File-western blot [file 41419_2022_5086_MOESM7_ESM.pdf]
