# Supplementary material for: Targeting HIC1/TGF-β axis-shaped prostate cancer microenvironment restrains its progression
Source: Cell Death Dis. 2022 Jul 19;13(7):624. doi: 10.1038/s41419-022-05086-z (PMC9296670; doi:10.1038/s41419-022-05086-z)
Supplement: Supplementary file 10 — Supplemental Figure legend and materials [file 41419_2022_5086_MOESM10_ESM.docx]

**Supplemental Fig. S1. Tissue-specific knockout of *Hic1* in mouse prostate**

(a) PCR genotyping of *PB-Cre; Hic1^Flox^* and *Pten^Flox^* mouse. All genotyping was performed by PCR using genomic DNA isolated from the tail tip of 3-to-4-week-old mice. The PCR bands detected in *Pten*-deletion mice or *Hic1*-deletion mice were of the predicted size, confirming that *Cre*-mediated excision of the exons.

(b-c) WB analysis show *Pten* and *Hic1* deletion is complete and prostate-specific, no ectopic *Pten* or *Hic1* deletion be detected in other tissues.

(d) Representative images of HIC1 IHC staining of prostate at 17 weeks. The HIC1 expression is intact in *Ctrl* mice, while the expression is lost in *dCKO* mice. Scale bars: 50 μm.

(e) Macroscopic image and IHC staining of AR and CK8 of the para-aortic lymph nodes (LNs) dissected form from *Ctrl* and *dCKO* mice at 20 weeks old. Scale bars: 50 μm.

(f) Representative images of AR IHC staining of para-aortic lymph nodes at 40 weeks old in *dCKO* mice. Scale bars: 50 μm.

(g) Representative images of HE and AR IHC staining of prostate at 40 weeks old mice. Scale bars: 200 μm.

(h-i) Representative images of HE and AR/Nkx3.1 or Ki-67 IHC staining show PCa cells spread into para-aortic lymph nodes or adrenal gland at 12 months old of *dCKO* mice. Scale bars: 50 μm.

(j) Representative images of IHC staining of CD163 and CD206 of the prostate at 17 weeks old mice. Scale bars: 50 μm. Red arrow indicate the macrophages in the stroma.

(k)Scatter dot plot shows quantitative analysis of the infiltrated M2 macrophages in stroma (n=5 each group, **P* < 0.05, Mann-Whitney U-test).

**Supplemental Fig. S2. HIC1 deleted-PCa cells induce the migration and polarization of M2 macrophages**

(a) CRISPR-Cas9-mediated HIC1 deletion in PC3 and C4-2B PCa cell lines. Cell lysates were analyzed by western blot analysis with antibodies against HIC1 and GAPDH. SgHIC1-3 and sgHIC1-4 means two different interferences of sgRNA.

(b) Schematic of xCELLigence RTCA-DP System to real-time monitor the migration capacity of RAW264.7.

(c) The curves of cell index represents motility capacity of RAW264.7 induced by PC3 Ctrl/ PC3 sgHIC1-4 or C4-2B Ctrl/ C4-2B sgHIC1-4 ( *****P*＜0.0001, *P* values were obtained using two-way ANOVA).

(d) Isolation of CD14^+^ PBMCs using CD14-beads from peripheral blood and FACS verification after labeled with CD14-FITC. Results show after CD14-beads enrichment, most PBMCs are CD14- positive.

(e) A co-culture system was used to test the polarization situation of macrophages induced by PCa cells.

(f) Percent of M2 macrophages induced by co-culture with PCa cells was showed in column charts (**P*＜0.05;***P*＜0.01. two-tailed Student’s *t*-Test) .

(g) FACS analysis show RAW264.7 cells are markedly M2 polarized when co-cultured with HIC1-deleted PC3 or C4-2B cells.

(h) Western blot analysis of RAW264.7 cell lysates was performed using antibodies against phosphorylation of STAT3 (Y705) (p-STAT3), STAT3 and GAPDH after activating by supernatant derived from HIC1-deleted PCa cells for 12 h.

(i) Comparation the HIC1 expression level of prostate cancer tumor tissue with adjacent normal prostate tissue using the TCGA+GTEx and GSE40272 dataset.

(j-k) The higher TGFB1 expression level correlates with lower progression-free survival in TCGA Prostate Cancer (PRAD) dataset as well as lower overall survival ratio in TCGA Pan-Cancer (PANCAN) dataset by Kaplan-Meier analysis.

**Supplemental Fig. S3. TGFB1 is a direct target of HIC1**

(a) HIC1 directly suppresses the TGFB1 promoter activity. Transient transfection of cells with HIC1 markedly inhibited TGFB1 promoter activity in a dose-dependent manner in 293T and PC3 cells.

(b) In-Silico analysis shows HIC1 has specific binding peaks in the TGFB1 promoter region in the 293T cell line, which consistent with our experimental findings.

**Supplemental Fig. S4.** **TGF-β promotes** **polarization of M2 macrophages and upregulates its *CXCR4* expression**

(a) Using the xCELLigence RTCA-DP System to real-time monitor the TGF-β induced the M2 macrophage migration ability. The migration of macrophages markedly increased after TGF-β treatment in 48 h compared with the control, which was sustained obviously until 72 h.

(b) M2 markers were determined by flow cytometry after treatment with TGF-β. IL-4 treatment in inducing M2 polarization as a positive control. The M2 polarization was markedly suppressed when treating with Galunisertib.

(c) qPCR analysis shows the TGFBR1 mRNA expression level was greatly upregulated in RAW264.7 cells, while the TGFBR2 was unchanged after TGF-β treatment.

(d) Western blot analysis of RAW264.7 cell lysates was performed using antibodies against phosphorylation of Smad2/3 (phospho S423 + S425) (p-Samd2/3), Smad2/3 and GAPDH after treatment with TGF-β treatment within 30 min, and this pathway was greatly inhibited by Gal treatment.

(e-f) TGF-β treatment upregulate CXCR4 expression in CD14^+^ PBMCs and RAW264.7 cell line in vitro. Expression of CXCR4 in these cells was verified at the mRNA and protein levels by flow cytometry(e), qPCR and western blot analysis (f). This effect was greatly inhibited by Gal treatment (e).

(g) After TGF-β treatment in both 24h and 48h, the increased CXCR4 expressions were inhibited by Gal treatment, followed by the decreased TGF-β receptor I expression.

(h) Immunofluorescence staining found M2 macrophages (CD206^+^, Green) in the stroma are co-expressed p-Smad3 (purple) and CXCR4 (red). Scale bar: 50 μm.

(i) Scatter dot plot show quantitative analysis of the infiltrated M2 macrophages co-expressed CXCR4 and p-Smad3 in the stroma markedly increased in the *dCKO* mice compared with the control group (n=3 each group, **P*＜0.05;****P*＜0.001. two-tailed Student’s *t*-Test).

**Supplemental Fig. S5.** **M2 macrophage** **secrete higher CXCL5 upon crosstalk with CAFs which in turn to promote PCa cell** **invasion and metastasis *via* EMT**

(a)NF (inactivated fibroblasts) and CAF (activated-fibroblasts) are isolated from inpatients who underwent radical prostatectomy (n =6) or prostatectomy of hyperplasia (n =6), respectively. The activation or inactivation status of fibroblast was determined by western blot analysis using antibodies against α-SMA and GAPDH.

(b) ELISA analysis found CAF1 has a higher CXCL12 than NF1 and NF2 (n=3 each group, *****P*＜0.0001, two-tailed Student’s *t*-Test).

(c) CD14^+^ PBMCs secrete higher CXCL5 after treatment with CXCL12 or co-cultured with CAF1, while this effect was derogated by CXCL12 neutralizing antibody (n=3 each group, *****P*＜0.0001, two-tailed Student’s *t*-Test).

(d) Scatter dot plot show quantitative analysis of invading and metastasis cells after treatment with or without rhCXCL5. This EMT process can be partial inhibited by SB225002.

(e) Upper panel: IHC analysis shows stronger CXCR2 staining intensity in the prostate of *dCKO* mice. lower panel: CXCR2 expression in PC3 cell line and prostate cancer patients with the aggressive phenotype. NG: negative control. GS: Gleason Score. Scale bar: 50μm (upper panel) and 20μm (lower panel).

(f) qPCR analysis shows the mRNA expression level of *CXCR2* was significantly knockdown in PC3 cells when using small interfering RNA (siRNA) (n=3 each group, *****P*＜0.0001, two-tailed Student’s *t*-Test).

(g) Comparation CXCR2 expressions level in metastatic tumor tissues with the primary tumor tissue using the GSE6919 dataset (Mann-Whitney U-test).

**Supplemental Methods**

**Primers for PCR-based genotyping of transgene mice**

Pb-Cre forward: 5'- CGGTCGATGCAACGAGTGAT-3'

Pb-Cre reverse: 5'- CCACCGTCAGTACGTGAGAT-3'

Pten forward: 5'- CAAGCACTCTGCGAACTGAG -3'

Pten reverse: 5'- AAGTTTTTGAAGGCAAGATGC -3'

Hic1 forward: 5'-CCCCACCTTTCTACACCTCA -3'

Hic1 reverse: 5'-GAGAGGCAGGGTTCTCCTTT-3'

**Co-culture**

To assay the effect of PCa cells on CD14^+^ PBMCs and Fibroblasts, PC3/C4-2B cells (3x10^5^) were added into the upper chamber and CD14^+^ PBMCs and fibroblasts (5x10^4^) were added into the lower chamber of a 6-well cell culture insert with 0.4 μm pore size (Cat. No. MCHT06H48, Millipore). Selected co-cultures were treated neutralizing antibodies to TGF-β (Cat. No.369, Abcam, Cambridge, UK) at 1 μg/mL. Co-culture lasted 5 days before subsequent western blotting and flow cytometry.

**Western blot analysis and antibodies**

Protein extracts were resolved through 8%-15% SDS-PAGE, transferred to PVDF membranes, blocked by 5% normal fetal bovine serum and probed with primary antibodies. Peroxidase-conjugated anti-mouse or rabbit antibody (Cat.No. [115-035-003](https://www.jacksonimmuno.com/catalog/products/115-035-003) and 111-035-003; Jackson Immumoresearch, PA) was used as secondary antibody and the antigen-antibody reaction was visualized by enhanced chemiluminescence assay (High-sig ECL Western Blotting Luminlo/Enhancer Solution, Cat. No. 180-5001; Tanon, Shanghai, China).

The primary antibodies are listed the below: GAPDH (Cat. No.60004-1-Ig; Proteintech, Wuhan, China), PTEN (Cat. No. 9188, CST, Shanghai, China), HIC1 (human: Cat. No. H8539, Sigma, St. Louis, MO; mouse: Cat. No. bs-15485R; Bioss), p-STAT3 (Cat. No. ab76315; Abcam), STAT3 (Cat. no. ab109085; Abcam), c-Myc (Cat. No. ab32072; Abcam), Lamin B1 (Cat. No. 66095-1-Ig, Proteintech), p-Smad2/3 (Cat. No. ab63399; CST), Smad2/3 (Cat. No. 8685; CST), CXCR4 (Cat. No. ab124824; Abcam), TGFBRI (Cat. No. ab31013; Abcam), p-Akt (Cat. No. 4060S; CST), Akt (Cat. No. 4691S; CST), N-cadherin (Cat. No. ab76011; Abcam), Vimentin (Cat. No. 5741; CST), Slug (Cat. No. ab27568; Abcam), CXCR2 (Cat. No. ab14935; Abcam)

**Nuclear and Cytoplasmic Extraction**

Nuclear and cytoplasmic proteins of RAW264.7 cells were prepared using nuclear and cytoplasmic extraction kit (Cat. No. 78833; Thermo Scientific, Shanghai, China) according to the manufacturer’s instruction.

**RNA extraction and quality control**

Total RNA was isolated from the tissues using TRI Reagent (Cat. No. T9424; Sigma) according to the manufacturer’s protocol. RNA quantity and quality were measured with NanoDrop ND-1000 (Thermo Scientific). RNA integrity was assessed by standard denaturing agarose gel electrophoresis.

**Quantitative real-time reverse transcription PCR**

RNA from each sample was reverse transcribed to cDNA using Prime Script^TM^ RT Master Mix (Cat. No. RR036A; TAKARA, Osaka, Japan). Quantitative real-time reverse transcription PCR(q RT-PCR) was performed with an LightCycler 480Ⅱ Real-Time PCR System (Roche, Mannheim, Germany), using the TB Green^TM^ Premix Ex Taq^TM^ (Cat. No. RR420A; TAKARA) according to the manufacturer’s instruction. Primers were designed using Primer 3 (http:// sourceforge.net/projects/primer3/) and obtained from GENEWIZ. Following an initial denaturation at 95 °C for 30 sec, 40 cycles of PCR amplification were performed at 95 °C for 5 sec and 60 °C for 20 sec. The levels of gene expression were quantified based on the cycle threshold (Ct) values and normalized to the internal control gene GAPDH or β-actin. The primer sequences are as following:

**Human:**

HIC1 forward: 5'- GTCGTGCGACAAGAGCTACAA-3'

HIC1 reverse: 5'-CGTTGCTGTGCGAACTTGC-3'

TGFB1 forward: 5'- *ACAACCGCACTGTCATTCAC*-3'

TGFB1 reverse: 5'- CGCCACTTTCCTCTCCAAAC-3'

TGFBR1 forward: 5'- GCAAAGGTCGATTTGGAGAA -3'

TGFBR1 reverse: 5'- TCAAAAAGGGATCCATGCTC -3'

TGFBR2 forward: 5'- AACGGTGCAGTCAAGTTTCC -3'

TGFBR2 reverse: 5'- GCACTTTGGAGAAGCAGCAT -3'

CXCR2 forward: 5'- AAGGTGGAAGTGGTAGCCTC -3'

CXCR2 reverse: 5'- CCTTCTTGTCTTCCCTGGGT -3'

GAPDH forward: 5'- CCATGTTCGTCATGGGTGTGAACCA -3'

GAPDH reverse: 5'- GCCAGTAGAGGCAGGGATGATGTTC -3'

**Mouse:**

CXCR4 forward: 5'- AACACGAGGATGGCAAGAGA -3'

CXCR4 reverse: 5'- GAGTCATAGTCCCCTGAGCC -3'

C-Myc forward: 5'- GGAGATCCGGAGCGAATAG -3'

C-Myc reverse: 5'- CCTTGCTCGGGTGTTGTAAGT -3'

β-actin forward: 5'- CACGATGGAGGGGCCGGACTCATC -3'

β-actin reverse: 5'- CCAAGGCCAACCGCGAGAAGATGAC -3'

**Isolation of primary PBMCs and fibroblasts**

Peripheral blood samples were collected from healthy adult volunteers by tubes containing acid-citrate-dextrose anticoagulant. Then PBMCs were isolated by using HISTOPAQUE (Cat. No. 10771, Sigma) gradient centrifugation (3000rpm, 10min). CD14^+^ PBMCs were isolated by human CD14^+^ microbeads，MACS columns and MACS separators according to the protocols(Cat. No. 130-050-201; MACS, Germany).

According to the protocol in our research before([1](#_ENREF_1)), primary cancer-associated fibroblasts of prostate were isolated from patients treated with radical prostatectomy and normal-associated fibroblasts of prostate were isolated from bladder cancer patients treated with radical surgeries. All fibroblasts used for experiments were cultured within 7 generations. The use of the clinical samples was approved by Fudan University Shanghai Cancer Center Ethical Committee, an approved and signed Institutional Review Board informed consent form was obtained from each participant (Shanghai, China).

**siRNAs transfection**

The 293T, RAW264.7 and PCa cells were transfected with Lipofectamine^®^ 3000 (Invitrogen, Carlsbad, CA) in serum-free Opti-MEM (Gibco, Carlsbad, CA) according to the manufacturer’s instruction. All siRNAs were synthesized from Biomics (Shanghai, China).

The strands of siRNAs were as follows:

**Human:**

Myc si: 5'- AACGUUAGCUUCACCAACAUUdTdT-3'

CXCR2 si-1: 5'- GGCAACAAUACAGCAAACUdTdT-3'

CXCR2 si-2: 5'- GUCUACUCAUCCAAUGUUAdTdT-3'

**Mouse:**

Myc-si-1: 5'- GGACACACAACGUCUUGGAdTdT-3'

Myc-si-2: 5'- CGACUCCGUACAGCCCUAUdTdT-3'

Myc-si-3: 5'- GAGUCGGGCUCAUCUCCAUdTdT-3'

**Construction of lentiviral vectors**

The Cas9 expression constructs Lenticas9-blast and the sgRNA expression plasmid Lentiguid-puro was used to generate stable HIC1 knockout PC3 and C4-2B cell lines. These plasmids were generated and preserved by our group^1^.

For generation of a stable *TGFB1* knockdown PC3 and C4-2B cell lines, GV248 lentiviral vectors expressing short hairpin RNAs targeting *TGFB1* were purchased from GeneChem company (Shanghai, China). Lentiviruses were produced and infected cells were selected by GFP. Three shRNAs (clone ID: TGFB1-RNAi-17673, 17674 and 17675) were used in the experiment, namely sh TGFB1-1, 2 and 3.

| **ID** | **Target sequence** | **Site** | **GC(%)** |
| --- | --- | --- | --- |
| shTGFB1-1 | TGCGGATCTCTGTGTCATT | 2154 | 47.37 |
| shTGFB1-2 | CGCGTGCTAATGGTGGAAA | 1193 | 52.63 |
| shTGFB1-3 | GGCCTTTCCTGCTTCTCAT | 1606 | 52.63 |

**Luciferase reporter assay**

Constructs of the *TGFB1* promoter region at -900/+840, -600/+840, -300/+840 and -10/+840 were synthesized from Obio Technology (Shanghai, China). *TGFB1* promoter construct pGL3-300/+840 contains three HIC1-responsive elements (HiREs), all of which were mutated (TGCC→GATT, named as M1, M2and M3) by Obio Technology. 293T and PCa cells were cultured in DMEM containing 10% FBS and transfected by Lipofectamine™ 3000 Reagent in 24-well plates. The *HIC1* plasmid: *TGFB1* promoter constructs (or mutant constructs): Renilla plasmid DNA (200ng: 200ng: 20ng) were co-transfected for 12 h and then incubated for 48h in fresh complete medium. Cells were then washed by cold PBS and lysed with the luciferase assay buffer. Luciferase activities were measured by using a dual luciferase assay kit (Cat. No. E1960; Promega, Madison, WI) with a chemiluminometer (Synergy H1 microplate reader, Biotek). Luciferase reporter assays of *CXCR4* promotor and its mutant constructs (E-box of c-Myc: CACCTG→TGTTCA, named as Mut1 and Mut2) were performed similar with *TGFB1* promoter. The results were expressed as ratio of firefly luciferase activity to Renilla luciferase activity. Data were analyzed as the mean values and standard deviations from at least three independent transfections performed in triplicate.

**Chromatin immunoprecipitation (ChIP)**

In brief, formaldehyde was added directly to the cultured PCa, 293T or PBMC cells in 10 cm plates to a final concentration of 1% for 10 min at room temperature. The cross-linking was stopped by adding glycine to a final concentration of 0.125 M for 5 min at room temperature. Cells were lysed directly in the plates by resuspension in 1 mL cell lysis buffer for 10 min on ice. Then, the samples were pelleted and resuspended in 1 mL nuclear lysis buffer, and sonicated to obtain chromatin fractions from 200bp to 1000bp using a BioRuptor (Diagenode, Liege, Belgium) on ice. After preclearing with a 50% slurry of protein A-G beads preincubated with salmon sperm DNA and bovine serum albumin for 4-6 h, at 4 °C, the chromatins were incubated with 5ug antibody (HIC1 or c-Myc) or 1 ug normal rabbit IgG overnight. The antibody bound chromatin was then pulled down for 3 h with protein A-G beads, washed extensively (low salt wash buffer, high salt wash buffer, LiCl wash buffer, and TE wash buffer) and eluted two times with Elution buffer. After addition of 8 μL of 5M NaCl, the cross-linking was reversed by 4 h (or overnight) incubation at 65 °C. The immunoprecipitated DNAs as well as whole cell extract DNAs (Input) were preliminarily purified by treatment with RNase A and then proteinase K followed by further purification. The purified DNA was used for real-time PCR analyses using the relevant primers for *TGFB1*, *CXCR4* and *GAPDH* following real-time PCR protocols as before. Special primers for *TGFB1*, *CXCR4* promotor were designed at the binding region with the transcription factors. Then the real-time PCR products were collected for standard denaturing agarose gel electrophoresis. A control primer specific for the human *GAPDH* promoter region was used for monitoring the experiment. Antibodies: HIC1 (human: Cat. No. H8539; Sigma), c-Myc (Cat. No. ab32072; Abcam).

The primer sequences are as following:

*GAPDH* promoter forward: 5'-TACTAGCGGTTTTACGGGCG-3'

*GAPDH* promoter reverse: 5'-TCGAACAGGAGGAGCAGAGAGCGA-3'

*TGFB1* promoter forward: 5'- GGAGGCAGCACCCTGTTTGCG-3'

*TGFB1* promoter reverse: 5'- GCTGGGCCACCGTCCTCA -3'

*CXCR4* promoter forward: 5'- GCCAAATCCTACCTTCTTCTG -3'

*CXCR4* promoter reverse: 5'- CTTCCTTCGGAGGATGTAGC -3'

**Supplementary Refrences**

1. Wang Y, Weng X, Wang L, Hao M, Li Y, Hou L*, et al.* HIC1 deletion promotes breast cancer progression by activating tumor cell/fibroblast crosstalk. The Journal of clinical investigation 2018;128:5235-50
